# Growth Hormone–Releasing Peptides: Investigation of Their Secondary Structure, Thermal Stability, and Model Membrane Interactions

**DOI:** 10.1002/chir.70083

**Published:** 2026-01-19

**Authors:** František Králík, Anna Kvíčalová, Adriana Salaďáková, Martin Kuchař, Vladimír Setnička

**Affiliations:** ^1^ Department of Analytical Chemistry University of Chemistry and Technology Prague 6 Czech Republic; ^2^ Forensic Laboratory of Biologically Active Substances and Department of Chemistry of Natural Compounds University of Chemistry and Technology Prague 6 Czech Republic

## Abstract

Growth hormone–releasing peptides (GHRPs) comprise a group of small synthetic peptides that can effectively influence growth hormone secretion both in humans and animals. As many health conditions are associated with growth hormone dysregulation, this class of compounds seems to be good candidates for various therapeutic purposes. However, GHRPs are also associated with doping in professional sports and they have been abused by amateur sportsmen and bodybuilders as well. In the present work, we investigated eight GHRPs by electronic circular dichroism (ECD) spectroscopy, which is inherently sensitive to the secondary structure of peptides and proteins. We stressed similarities and differences in their ECD spectra with respect to the similarities and differences of their respective chemical structures. We also studied interactions of the selected compounds with a model membrane system consisting of sodium dodecyl sulfate (SDS) micelles. The most interesting ECD spectral changes were observed for the GHRP‐5—SDS micelles system, where the induced ECD signal indicated the formation of α‐helical‐like secondary structure. To address the observed phenomena, conformational search with subsequent ECD spectra calculation at the time‐dependent density functional theory (TD‐DFT) level was performed to clarify the secondary structure changes. To the best of our knowledge, this is the first work where such a broad ensemble of GHRPs was systematically studied by ECD and the achieved results document that this approach provides a suitable analytical tool not only for a description of their natural conformational preferences, but can also bring insight into their possible interactions with the surrounding environment.

## Graphical abstract

Eight growth hormone–releasing peptides were analyzed by electronic circular dichroism spectroscopy, including evaluation of their thermal stability and interactions with model membranes. In the selected case, occurring interactions were successfully described by the density functional theory calculations, revealing how model membranes affected conformational preferences.

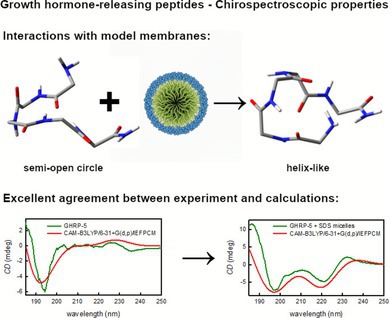

## 1 Introduction

Growth hormone secretagogues (GHSs) regulate the secretion of the growth hormone (GH) somatotropin in the human body. Growth hormone–releasing peptides (GHRPs) generally designate a group of small synthetic peptides (most commonly penta‐, hexa‐, or heptapeptides) that enhance the release of the GH. The natural GHRP, ghrelin, is a Ser^3^‐octanoylated peptide consisting of 28 amino acids and was first isolated and described in 1999 [1]. It is interesting to note that synthetic GHRPs had been discovered before the natural ones, which was part of the so‐called reversed pharmacology approach, where synthetic GHSs had helped discover the GH secretagogue receptor and the natural GHS ghrelin was identified and isolated thereafter [2, 3, 4, 5, 6]. The first synthetic GHRP with confirmed in vivo and in vitro GH‐releasing activity was hexapeptide GHRP‐6 in 1984, which is also one of the studied compounds in this work [3, 7]. Many health conditions such as benign prostate hyperplasia [8], Cushing's disease [9] and various cardiovascular diseases [10, 11, 12] have been associated with a dysregulation of GH secretion, and new methods of effectively controlling GH regulation have been searched for over the last few decades, and the potential therapeutic use of the synthetic GHRPs was brought to the forefront [13, 14].

Due to their positive effects on muscle growth, GHRPs are unfortunately associated with doping in professional sports. They are present in the 2025 List of Prohibited Substances and Methods of World Anti‐Doping Agency in the subsection S2.2.4 Growth hormone–releasing factors [15]. For this reason, methods for their reliable identification and quantification in various kinds of biological matrices (plasma, urine) were developed, the most common being methods based on high‐performance liquid chromatography [16, 17, 18, 19].

The secondary structure of proteins and peptides is known to be closely related to their biological activity and electronic circular dichroism (ECD) is a well‐established, powerful tool for monitoring secondary structure preferences and induced changes [20, 21]. For instance, ECD along with nuclear magnetic resonance was successfully used for evaluation of the temperature effects on ghrelin and its truncated analogs [22]. In this work, the ECD spectra of eight different GHRPs (Table 1, the chemical structures can be found in Supplementary Information, Figure S1) were recorded and their typical spectral patterns were described. All the studied compounds have been proven to positively influence GH secretion, although their structures or amino acid sequences vary. With respect to this fact, we focused on a description of the differences and similarities in their ECD spectra as specific secondary structure preferences may be a key factor for the important GH regulatory properties. We also analyzed if interactions of the studied GHRPs with micelles formed by sodium dodecyl sulfate (SDS) occur. SDS micelles represent a simple membrane model with a hydrophilic surface and hydrophobic bulk and we used them as a preliminary testing system for monitoring potential conformational changes of GHRPs in their presence as similar interactions were successfully monitored by ECD for des‐acyl‐ghrelin and obestatin, both exhibiting a similar biological activity as GHRPs [23, 24]. To the best of our knowledge, this is the first work where such an extensive sample of GHRPs was subjected to the ECD analysis and the achieved results including monitoring of model membrane interactions document that the selected approach can bring further insight into biological activity mechanisms of this fascinating class of compounds.

**TABLE 1:** Analyzed GHRPs.

Compound	IUPAC name (sequence)	M (g·mol^−1^)
GHRP‐1	L‐Ala‐L‐His‐3‐(2‐Naphthyl)‐L‐Ala‐L‐Trp‐D‐Phe‐L‐Lys‐NH_2_	955.1
GHRP‐2	D‐Ala‐3‐(2‐Naphthyl)‐ L‐Ala‐L‐Trp‐D‐Phe‐L‐Lys‐NH_2_	818.0
GHRP‐3	α‐Methyl‐L‐Ala‐D‐Trp‐D‐Pro‐D‐Ile‐L‐Arg‐NH_2_	654.8*
GHRP‐4	D‐Trp‐L‐Ala‐L‐Trp‐D‐Phe‐NH_2_	607.7
GHRP‐5	L‐Tyr‐D‐Trp‐L‐Ala‐L‐Trp‐D‐Phe‐NH_2_	770.9
GHRP‐6	L‐His‐D‐Trp‐L‐Ala‐L‐Trp‐D‐Phe‐L‐Lys‐NH_2_	873.0
Ipamorelin	α‐Methyl‐L‐Ala‐L‐His‐3‐(2‐Naphthyl)‐D‐Phe‐L‐Lys‐NH_2_	711.9
Anamorelin	**	546.7

* GHRP‐3 was obtained as a TFA salt with an unknown molar ratio; the given *M* is related to a single molecule of GHRP‐3.

** Anamorelin does not have a common peptide sequence; its chemical structure can be found in Supplementary Information, Figure S1.

## 2 Materials and Methods

### 2.1 Materials and Sample Preparation

Anamorelin was provided by Scintila (Czech Republic), GHRP‐3 was provided by Toronto Research Chemicals (United Kingdom), and all the other peptides were purchased from Prospec Bio (Israel). For the ECD measurements of peptides, solutions were prepared by dissolving the given peptide in ultrapure distilled water to achieve the optimal concentration. GHRP‐5 and GHRP‐6 were measured at two concentrations to achieve reasonable values of absorbance (below 1.8) in the whole observed spectral region. The concentrations of all the measured GHRP solutions are presented in Table 2.

**TABLE 2:** Concentration of measured solutions of studied peptides in distilled water and in the presence of SDS micelles.

Compound	Concentration in aqueous solution (mg·mL^−1^)	Concentration with SDS micelles (mg·mL^−1^)
GHRP‐1	0.21	0.10
GHRP‐2	0.18	—
GHRP‐3	0.17	—
GHRP‐4	0.10	—
GHRP‐5	0.17/0.09*	1.00/0.05**
GHRP‐6	0.19/0.12*	—
Ipamorelin	0.16	0.04
Anamorelin	0.21	0.05

* The first concentration refers to the measurement in the 210‐ to 320‐nm region, the second concentration refers to the measurements in the 185‐ to 210‐nm region.

** The first concentration refers to the measurement in the 230‐ to 320‐nm region; the second concentration refers to the measurements in the 185‐ to 250‐nm region.

In order to study interactions of the peptides with SDS micelles, the solutions for measurements were prepared by dissolving the given peptide in a freshly prepared SDS solution with a concentration of 8.77 × 10^−3^ mol·L^−1^, which is above the critical micellar concentration. For the evaluation of GHRP‐5 interactions with SDS micelles, the concentrations of the peptides had to be adjusted to obtain reasonable signal intensities (absorbance below 1.8) in the spectral regions 185–250 and 250–320 nm (Table 2). In the case of GHRP‐1 and ipamorelin, the final concentration of the peptide had to be lowered to avoid aggregation, and the concentration of anamorelin in the presence of SDS micelles was lowered due to a significant enhancement of band intensities (Table 2).

### 2.2 Electronic Circular Dichroism and UV Absorption Spectra Measurements

The ECD and UV absorption spectra were measured on a J‐815 spectrometer (Jasco, Japan) in a 1‐mm quartz cuvette (Hellma, Germany) at a 20‐nm·min^−1^ scanning speed and 8‐s response time in the 320‐ to 185‐nm region. The final spectra were presented as an average of five measurements. The baseline correction was performed by subtracting the spectrum of distilled water or SDS solution measured under the identical experimental conditions. The temperature was controlled with a Jasco Peltier temperature control system CDF‐426S/16.

### 2.3 Conformational Search and ECD Spectra Simulation

The conformational search of GHRPs was performed by the conformer‐rotamer sampling tool (CREST) [25, 26] with the GFN2‐xTB method [27] and implicit solvation considered by the analytical linearized Poisson‐Boltzmann (ALPB) model [28]. Rotational strengths were calculated by the time‐dependent density functional theory (TD‐DFT) framework within the Gaussian 16 software package, Revision C.01 [29], at the CAM‐B3LYP/6‐31+G(d,p) level of theory with implicit solvation considered via the integral equation formalism version of the polarizable continuum model (IEFPCM). As an overwhelming amount of stable conformers was expected to be found by the CREST procedure, the TD‐DFT calculations were performed for the first 20 lowest‐energy conformers. The final calculated ECD spectra were obtained by assigning a Gaussian band shape with a 10‐nm half‐width at half‐maximum to the calculated rotational strengths.

## 3 Results and Discussion

### 3.1 Electronic Circular Dichroism

**FIGURE 1:**
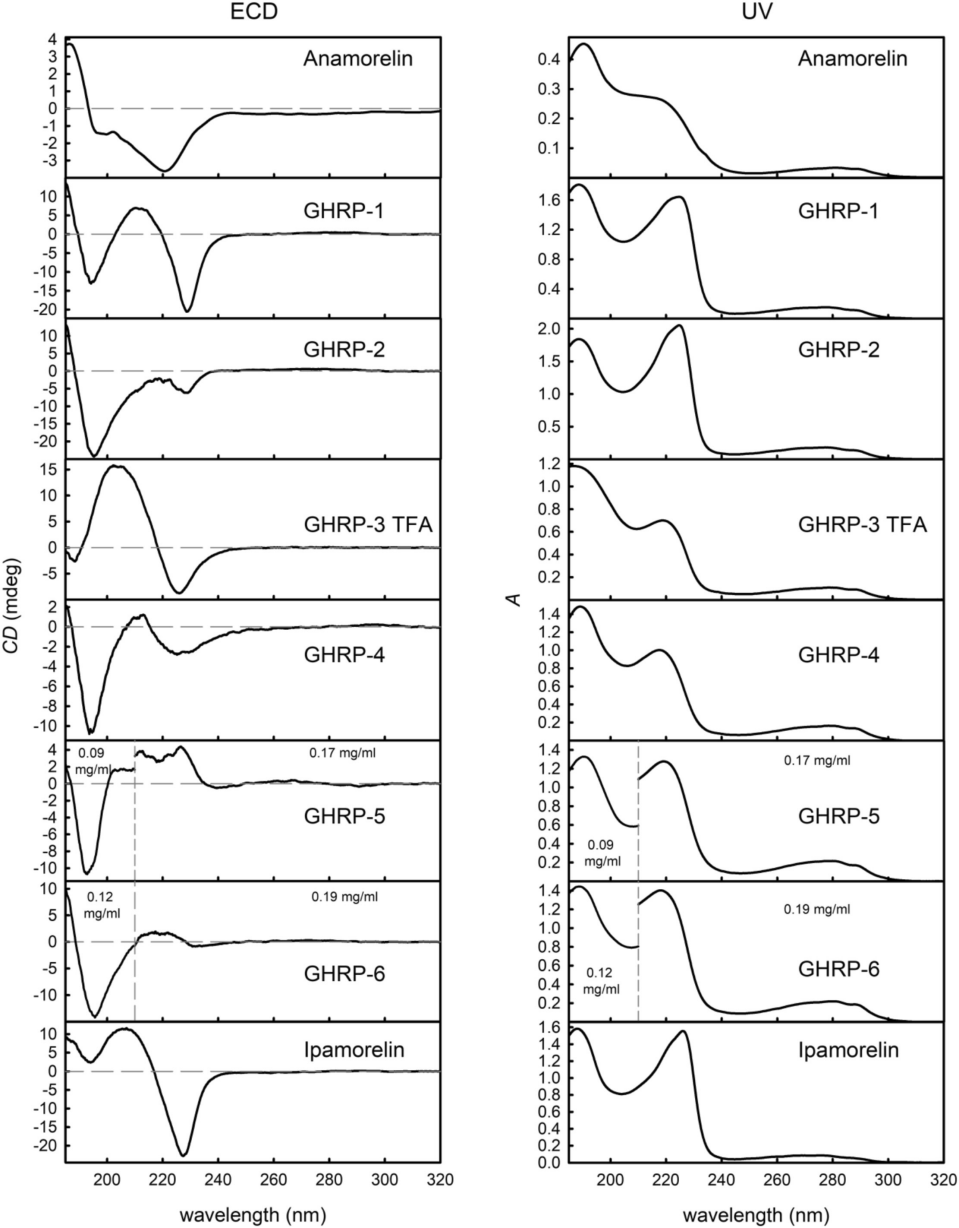
The ECD (left) and UV absorption spectra (right) of the studied GHRPs. GHRP‐5 and GHRP‐6 were measured with two different concentrations in the 185‐ to 210‐ and 210–320‐nm regions, respectively, to reach optimal absorbance values.

Similar spectral patterns can be observed in the UV absorption spectra (Figure 1 right, Table 3) of the studied GHRPs: a low intense broad band ranging ~240–300 nm and two bands with maxima at ~220–230 nm (*n*–π* transitions) and ~190 nm (π–π* transitions) [30]. In the case of GHRP‐1, GHRP‐2, and ipamorelin, a shoulder at lower wavelengths can be clearly recognized within the ~220‐ to 230‐nm bands. It is also worth noting that relative intensities of the two bands differ for individual GHRPs—the ~220‐ to 230‐nm band is more intense in the spectra of GHRP‐1, GHRP‐2, and ipamorelin when compared to the ~190‐nm band. This can be probably attributed to the presence of the naphthyl group in the structure of all three GHRPs, while it is not present in the structures of other peptides. On the other hand, the band at ~190 nm in the UV spectra of GHRP‐5 and GHRP‐6 exhibited the highest relative intensity (the measurements had to be conducted at lower concentrations in the spectral region in question in order to keep the absorbance values under a reasonable threshold), which can be caused by the double tryptophan and phenylalanine moieties.

**TABLE 3:** Maxima of the observed bands in the UV and ECD spectra of the studied GHRPs. The sign of the ECD bands is provided in parentheses.

Compound	UV bands (nm)	ECD bands (nm)
GHRP‐1	225, 189	228(−), 212(+), 194(−), < 185(+)
GHRP‐2	225, 189	~228(−), 195(−), < 185(+)
GHRP‐3	219, ~185	226(−), 202(+), 189(−)
GHRP‐4	218, 189	229(−), 213(+), 194(−), < 185(+)
GHRP‐5	219, 191	235–210(+), 193(−), < 185(+)
GHRP‐6	218, 189	244–230(−), 230–210(+), 196(−), < 185(+)
Ipamorelin	226, 188	227(−), 205(+), < 185(+)
Anamorelin	219, 190	221(−), 188(+)

The ECD spectra of the studied GHRPs (Figure 1 left, Table 3) show more variety than the UV absorption spectra. A negative band in the ~220‐ to 230‐nm range can be observed in the ECD spectra of all GHRPs except GHRP‐5 and GHRP‐6. In the GHRP‐5 ECD spectrum, a low‐intensity positive broad band with uncertain maximum position can be observed in this region, while a very weak negative Cotton effect‐like signal is present in the GHRP‐6 spectrum. Although GHRP‐5 and GHRP‐6 have very similar UV absorption spectra, their ECD exhibits significant differences to provide a good basis for their mutual recognition (the above‐mentioned differences in the 220‐ to 230‐nm region and the negative band in the lower wavelength region is significantly broader in the GHRP‐6 spectrum). A positive band with varied intensities and maxima positions follows in the ECD spectra of GHRP‐1, GHRP‐3, GHRP‐4, and ipamorelin, and in the region below 200 nm, all compounds except anamorelin and GHRP‐3 have a negative band with a maximum at ~195–198 nm followed by a partially present positive band with further decreasing wavelength. It is not possible to measure below 185 nm due to the absorption of water; however, the data reliably document that a positive band can be expected there. In the ECD spectrum of anamorelin, the negative band with a maximum at 222 nm is significantly broader when compared to other studied GHRPs, and it is directly followed by a positive band with a maximum at 188 nm. GHRP‐2, on the other hand, does not provide any positive band in the 200‐ to 230‐nm region. Finally, ipamorelin has only two positive bands in the lower wavelength region, one having a maximum at 208 nm, and the second one goes below 185 nm as in the case of the other GHRPs.

After taking a closer look at the chemical structures of the studied GHRPs, two pairs of very similar compounds can be identified: GHRP‐1 and GHRP‐2, and GHRP‐5 and GHRP‐6. Correspondingly, also their UV and ECD spectra share similarities—the UV spectra of GHRP‐1 and GHRP‐2 are almost the same except for the difference of the relative intensities of the two dominating bands. In the ECD spectrum of GHRP‐1, however, the negative band at 228 nm is much more intense and better resolved than in the spectrum of GHRP‐2, and the following positive band at 212 nm is absent in GHRP‐2’s spectrum. These differences have to be attributed to the L‐Ala‐L‐His terminal substitution in GHRP‐1’s structure, which is the only structural difference in comparison to GHRP‐2, where D‐ala is present. As the UV spectra of both compounds are very similar and the ECD spectra exhibit more differences, it is probable that the different substituents affect preferable conformations, which the two compounds adopt in aqueous solutions.

The UV and ECD spectra of GHRP‐5 and GHRP‐6 also share a lot of similarities. While it does not seem that any tangible differences can be identified in the UV spectra, the ECD spectra offer more variability—the intense negative band at 193/196 nm is obviously broader in the spectrum of GHRP‐6, and the broad bands with low intensities in the 245‐ to 210‐nm region also show differences. However, the differences do not seem to be as striking as in the case of GHRP‐1 and GHRP‐2, so it can be presumed that GHRP‐5 and GHRP‐6 naturally adopt more similar conformations. In contrast to the first case, the difference in the terminal substitution is L‐Tyr and L‐His for GHRP‐5 and GHRP‐6, respectively, which both contribute with an aromatic moiety, which can be the explanation of the more similar spectral patterns.

After taking a closer look at the lowest‐energy conformers of the four above‐mentioned compounds, it turned out that most of them have the indole ring of the tryptophan aligned along the phenyl ring of the phenylalanine, which indicates a π–π interaction (sample geometries can be found in the Supplementary Information, Figure S2). This stabilization can explain why the different substitutions at the other end of the molecules would have a higher impact on the preferred orientation of these substances in aqueous solutions.

The UV and ECD spectra of ipamorelin show some resemblance to the spectra of GHRP‐1, especially the UV spectrum. The similarity can be explained by the presence of similar structural moieties in the terminal parts of both molecules—GHRP‐1 has the same peptide sequence as ipamorelin with the inclusion of L‐Ala‐L‐Trp in the middle of the structure, and on the other hand, ipamorelin has an abundant α‐methyl group at the L‐Ala end.

Unsurprisingly, the UV and ECD spectra of anamorelin are unique compared to the other compounds as its chemical structure also differs significantly and lacks the typical peptide sequence nature. GHRP‐3 and GHRP‐4 have both different peptide sequences and other substituents than the other studied GHRPs, yet it is apparent that the ECD spectrum of GHRP‐3 is similar to the spectra of GHRP‐1 or ipamorelin if slight band shifts are taken into account. Similarities between the ECD spectra of GHRP‐4 and GHRP‐2 can be observed as well; however, the UV spectra of both GHRP‐3 and GHRP‐4 are distinct from the above‐mentioned compounds and most closely resemble the UV spectrum of anamorelin, which is interesting and a closer investigation of these compounds will be carried out in the future.

### 3.2 Thermal Stability

**FIGURE 2:**
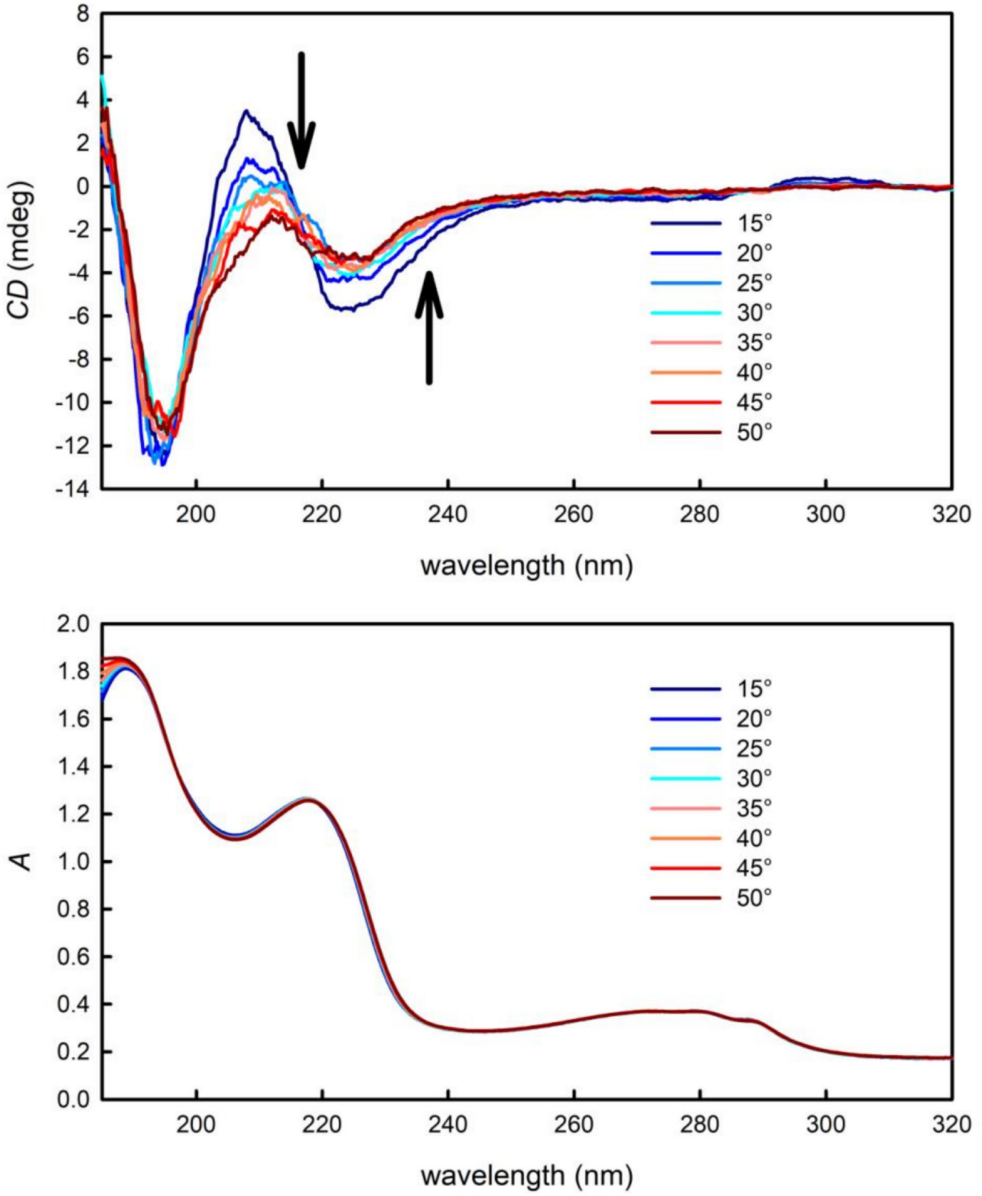
The temperature dependence of the ECD (top) and UV absorption spectra (bottom) of GHRP‐4.

For the selected GHRPs, effects of temperature on their secondary structure were investigated. Upon increasing the temperature of the GHRP‐4 solution, significant changes in the ECD spectra were observed (Figure 2 top): the intensities of both the negative band at 229 nm and the positive band at 213 nm decreased with increasing temperature, while such a strong effect was not observed in the spectral region below 200 nm. Also, a bathochromic shift in the maximum of the positive band at 213 nm is apparent with increasing temperature. It is worth noting that almost no changes were observed in the UV absorption spectra; thus, it can be concluded that increased temperature leads to changes in the GHRP‐4’s secondary structure. It is rather cumbersome to interpret the observed changes more precisely with respect to the specific secondary structure changes as the ECD spectra do not resemble any of the common secondary structure spectral patterns. That is not surprising considering that the investigated compounds are shorter, synthetic species.

**FIGURE 3:**
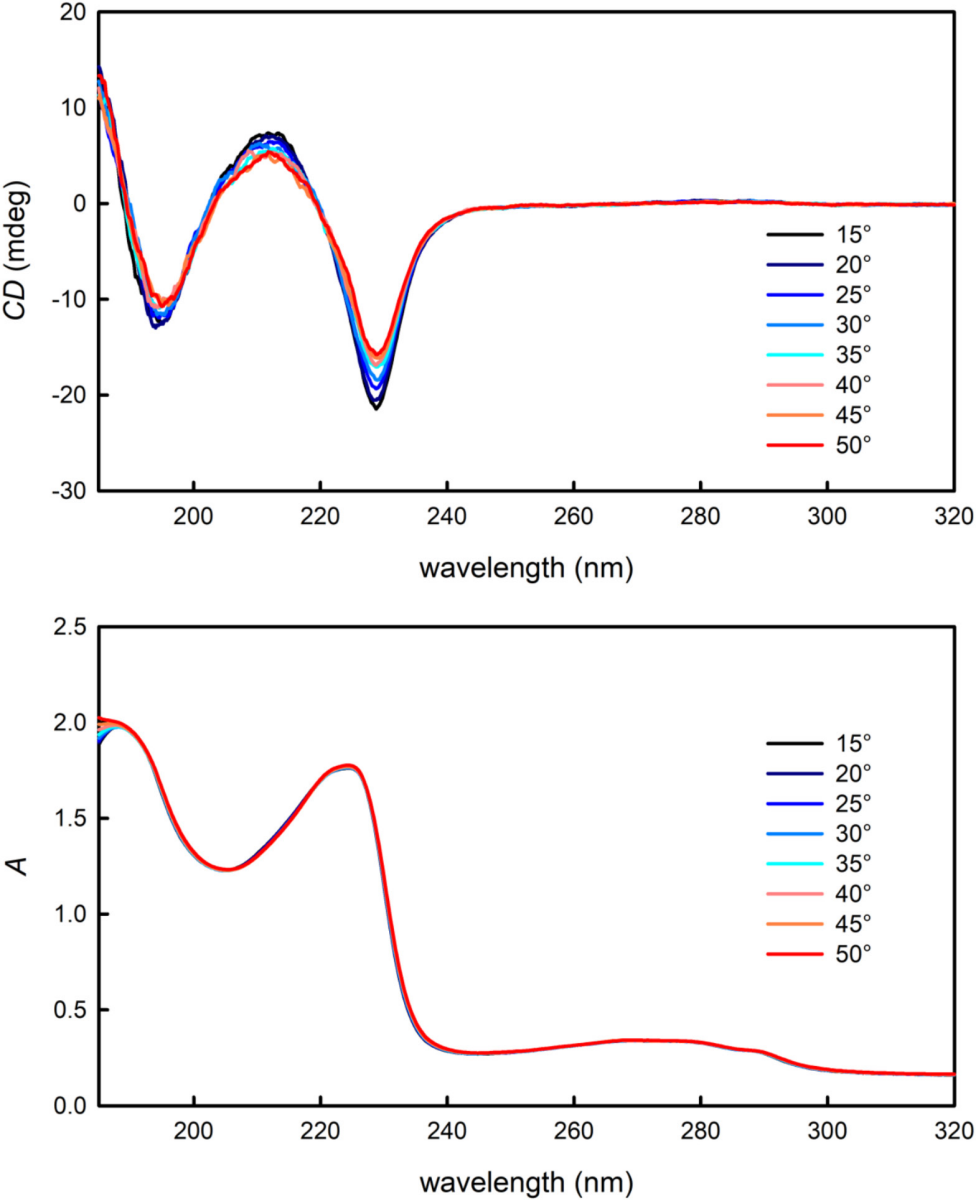
The temperature dependence of the ECD (top) and UV absorption spectra (bottom) of GHRP‐1.

The temperature dependence of the secondary structure was also studied for all the other compounds except anamorelin, but no significant changes in the UV and ECD spectra were observed. For this reason, only the results for GHRP‐1 are presented in the main text and the rest can be found in the Supplementary Information (Figures S3–S7). An overall slight decrease of band intensities with increasing temperature was observed in the ECD spectra of GHRP‐1 (Figure 3). In comparison to GHRP‐4, no spectral shifts or changes in band relative intensities were detected, and thus increasing temperature probably led only to a slight loosening of the secondary structure that GHRP‐1 forms naturally. No changes in UV absorption spectra were observed after changing temperature, and GHRP‐1 can be thus considered to be thermally stable.

### 3.3 Interactions of GHRPs With SDS Micelles

Micelles made of SDS with a hydrophilic surface and hydrophobic bulk were used as a simple membrane model and their interactions with GHRPs were studied. After SDS solution addition to GHRP‐2, GHRP‐3, GHRP‐4, and GHRP‐6, an immediate particle aggregation was observed and the solution could not be subjected to spectroscopic analysis. GHRP‐1 and ipamorelin formed turbid solutions after SDS micelles had been added, which indicated partial aggregation; after dilution, however, clear solutions were obtained. Anamorelin was the only compound where the presence of SDS micelles caused a significant enhancement of band intensities, and the solution had to be diluted as well to keep the absorbance values reasonable.

**FIGURE 4:**
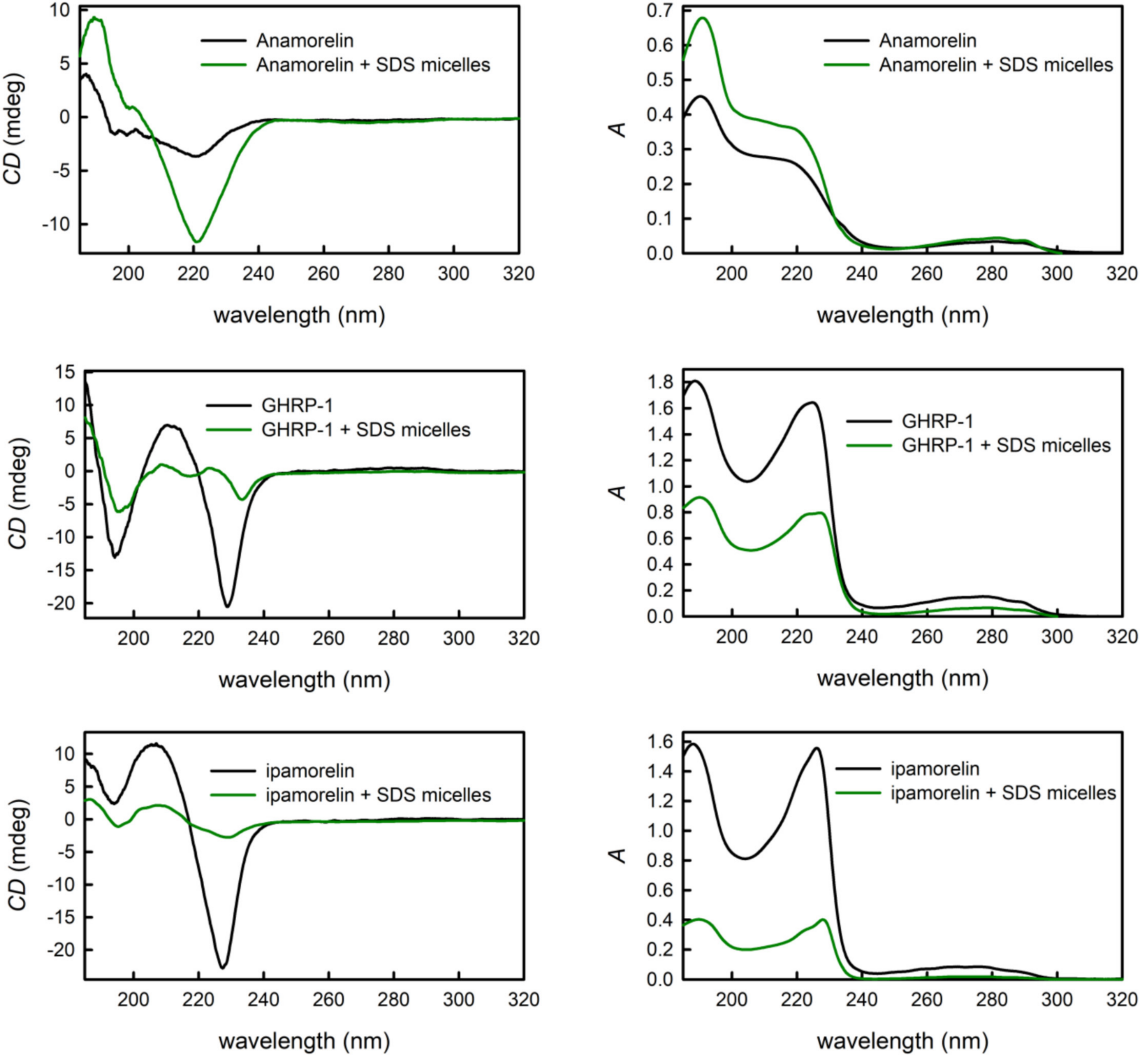
The ECD (left) and UV absorption spectra (right) of anamorelin (top), GHRP‐1 (middle) and ipamorelin (bottom) in aqueous solution (black line) and in the presence of SDS micelles (green line). The concentrations of a given GHRP were as follows (the first number refers to concentration in aqueous solution, the second number to the concentration in the presence of SDS micelles): anamorelin 0.21/0.05 mg·mL^−1^, GHRP‐1 0.21/0.10 mg·mL^−1^, and ipamorelin 0.16/0.04 mg·mL^−1^.

The UV absorption spectrum of anamorelin (Figure 4 top right) in the presence of SDS micelles does not differ from the spectrum of anamorelin aqueous solution as the same three bands with very similar relative intensities are present in both spectra. However, the absolute intensities of the two more intense bands in the lower wavelength region are surprisingly higher in the presence of micelles, although the concentration of anamorelin was more than four times lower than in the aqueous solution. Similar enhancement can be observed in the ECD spectra (Figure 4 top left), but the intensity increase of the negative band at 221 nm is higher than the intensity increase of the 188‐nm positive band. A slight bathochromic shift is also present for the positive band. The observed enhancement of the band intensities can be possibly explained by a stabilization of specific conformation(s) by the presence of SDS micelles. It is interesting that only anamorelin exhibits such a behavior in comparison to the other studied compounds; on the other hand, its chemical structure has a completely different nature and lacks the genuine peptide character, so different interactions with micelles can be expected. Cases of compounds of a similar size as anamorelin can be found in literature, where the molar extinction coefficients were also augmented by the presence of SDS micelles [31, 32].

The UV absorption spectrum of GHRP‐1 in the presence of micelles is also very similar to the UV spectrum of GHRP‐1 in aqueous solution (Figure 4 middle right). The lower intensities of the bands correspond to the difference in GHRP‐1 concentrations as the solution with micelles had to be diluted to avoid aggregation. The only difference is a slight bathochromic shift of the 225‐nm band. The ECD spectra, however, provided more distinct changes (Figure 4 middle left). Although the intensity decrease should be expected due to the concentration difference in the systems with and without micelles, other changes in the band composition can be observed in the 205‐ to 240‐nm region. A clearly resolved negative band at 227 nm and a positive band at 205 nm can be observed in the ECD spectrum of aqueous solution, while a negative band shifted to 233 nm and two very low intense positive bands with maxima at 223 and 209 nm appeared in the ECD spectrum of the solution containing micelles. It can be concluded that SDS micelles affect the secondary structure preferences of GHRP‐1.

Both the UV absorption and ECD spectra of ipamorelin in solution containing SDS micelles do not differ significantly from the spectra of ipamorelin in aqueous solution (Figure 4 bottom). There are clear differences in the band intensities; however, they correspond well to the difference in ipamorelin concentration. It can be concluded that the presence of SDS micelles probably does not affect the secondary structure of ipamorelin in a significant manner. On the other hand, another important conclusion is that SDS micelles did not cause aggregation as in the case of GHRP‐2, GHRP‐3, GHRP‐4, and GHRP‐6.

**FIGURE 5:**
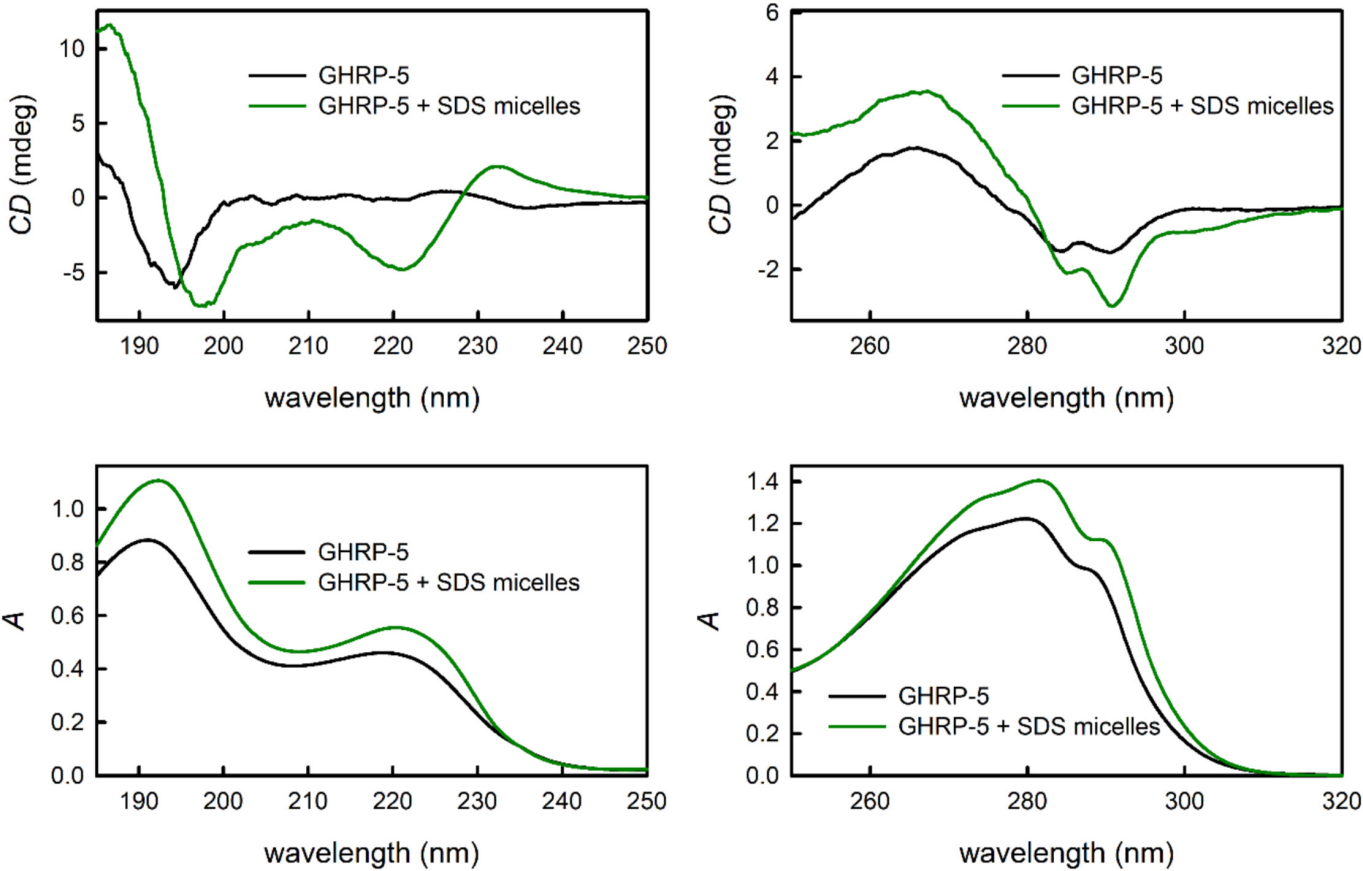
The ECD (top) and UV absorption spectra (bottom) of GHRP‐5 in aqueous solution (black line) and in the presence of SDS micelles (green line) measured at two different concentrations: 1.0 mg·mL^−1^ (right) and 0.05 mg·mL^−1^ (left).

The most intriguing spectral changes in the presence of SDS micelles can be observed for GHRP‐5 (Figure 5). In order to monitor changes in the 250–320‐nm region where GHRPs generally exhibit only very weak absorption bands, the concentration of GHRP‐5 was raised to 1.0 mg·mL^−1^ in both aqueous and SDS solution. The UV spectra in this region (Figure 5 bottom right) are almost identical; only small differences in absolute band intensities can be noticed. The changes in the ECD spectra (Figure 5 top right) are more prominent, especially the change in relative intensities of two negative bands in the 280‐ to 300‐nm region.

It is also worth noting that while GHRP‐5 was easily soluble in the solution containing SDS micelles, GHRP‐6, which has a very similar structure and also the UV and ECD spectra in aqueous solutions differed only slightly, immediately formed aggregated precipitates. After taking a closer look at the chemical structures of both compounds, the difference in the interaction should be caused either by the different aromatic substitution at the D‐Trp end (L‐His vs. L‐Tyr) or by the presence of lysine in the structure of GHRP‐6. GHRP‐1, GHRP‐2 and ipamorelin also contain lysine in their chemical structures and, interestingly, GHRP‐2 precipitated in the same way as GHRP‐6 after SDS addition, while GHRP‐1 and ipamorelin needed to be diluted in order not to precipitate from the solution. It is thus probable that the presence of lysine helps form aggregates/precipitates with SDS micelles.

The UV absorption spectra in the 185‐ to 250‐nm region (Figure 5 bottom left) also did not change significantly after the addition of SDS micelles. However, completely different spectral patterns can be observed in the ECD spectra (Figure 5 top left), where the presence of SDS micelles gave rise to the typical α‐helix bands: a negative one at 222 nm, a more intense negative band at 198 nm and a positive band at 187 nm. Although there is also a positive band with a maximum at 231 nm, which is not a typical pattern for α‐helical secondary structure, it can be concluded that the presence of SDS micelles causes GHRP‐5 to prefer—at least partially—conformations that resemble the classic α‐helix of naturally occurring peptides and proteins. In addition, the thermal and time stability of the GHRP‐5—SDS micelles system was confirmed (see Supplementary Information, Figures S8 and S9).

In order to interpret the induced changes in the ECD spectrum, a conformational search via CREST followed by ECD spectra calculations by TD‐DFT was performed. First, it has to be pointed out, however, that the goal of the calculations was not a precise description of GHRP‐5’s conformational preferences as this presents a very demanding task due to its size and most notably high flexibility. Furthermore, the effects of water solvent molecules were considered only implicitly, which usually does not provide sufficiently precise results as water can play an important role in various intra‐ and intermolecular interactions. The selected approach was based on the idea to find relevant stable conformers and among them search for structures that give predicted ECD spectra similar to those observed experimentally.

**FIGURE 6:**
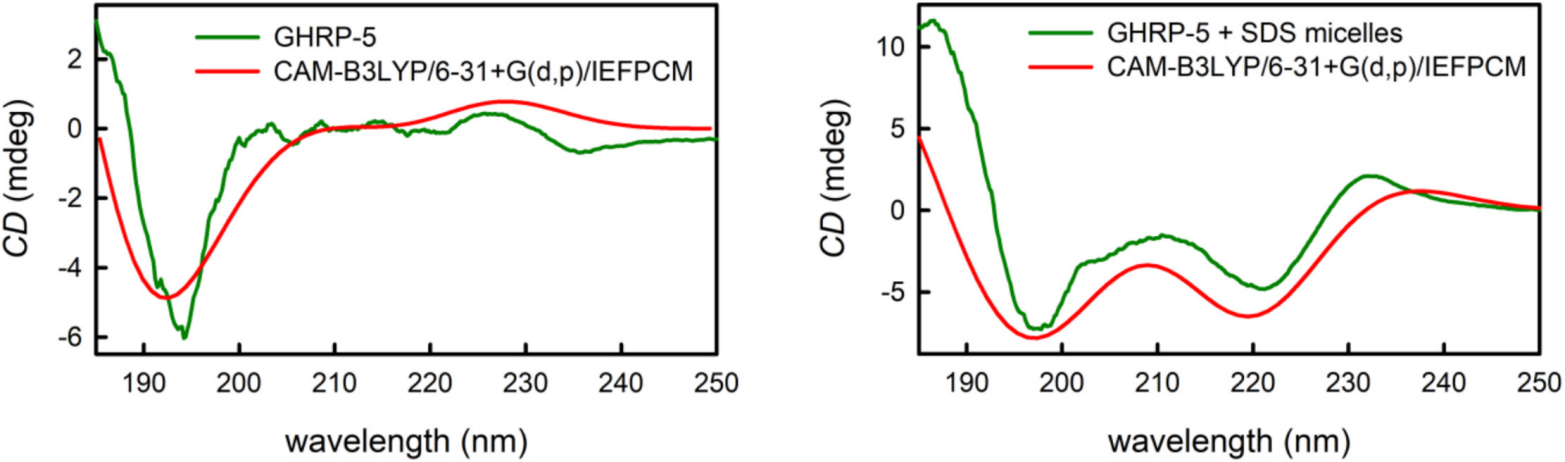
A comparison of the experimental ECD spectrum of GHRP‐5 in aqueous solution (left) and in the presence of SDS micelles (right) with the CAM‐B3LYP/6‐31+G(d,p)/IEFPCM calculated ECD spectra.

The CREST procedure revealed 453 stable conformers of GHRP‐5 and the 20 lowest‐energy ones were subjected to further ECD spectra calculations as the starting point for an evaluation of the selected approach. Of the 20 calculated ECD spectra, we were able to identify those closely resembling the experimental spectra both in aqueous solution and in the presence of SDS micelles. For this purpose, the shape‐fitting algorithm described in our previous work was used [33]. The ECD spectrum in the 185‐ to 250‐nm range of GHRP‐5’s aqueous solution is dominated by the single band at 193 nm, and the same spectral shape was predicted for two conformers (designated aq‐I and aq‐II), with the optimal abundances of 80 and 17% giving the closest match with the experiment, including the very weak positive band at higher wavelengths (Figure 6 left). It has to be noted though that the presented approach uses a purely mathematical comparison of the calculated and experimental spectra, and considering that the experimental spectrum contains a single dominant band, the conclusions derived by the applied algorithm can be misleading. After taking a closer look at the calculated ECD spectra of the other conformers (Figure S10), many of them also reach a reasonable agreement with the experimental spectrum, but their band maxima and/or relative intensities of the bands do not provide such a good agreement as the two conformers proposed by the shape‐fitting algorithm. It is also not probable that such a flexible system as GHRP‐5 would have only two dominant conformers. The achieved conclusion should be thus interpreted carefully and the two preferred conformers would almost certainly not play such a dominant role in GHRP‐5’s conformational preferences as predicted. Nevertheless, the achieved agreement and the fact that the conformers’ energies were predicted to be among the 20 lowest‐energy geometries lead to a conclusion that the two conformers would probably belong to the most preferred 3D structures that GHRP‐5 adopts. A possible danger of such pitfalls has been recently very well described in literature [34]. In the case of SDS micelles, where the ECD spectrum was significantly richer, it turned out that if the major contribution of three different conformers (65% of conformer mic‐I, 25% of conformer mic‐II and 8% of conformer mic‐III) is considered, an excellent agreement with the experimental spectra is achieved (Figure 6 right). The Cartesian coordinates of all five conformers can be found in Supplementary Information.

The most abundant conformer aq‐I, that was predicted for aqueous solutions, is stabilized by the hydrogen bond interactions between L‐tyrosine's carbonyl group with both the L‐tryptophan and L‐alanine amide groups, which causes the backbone to take a planar semicircular shape. On the other hand, the structure of the second conformer aq‐II is stabilized by two hydrogen interactions: between the D‐tryptophan's carbonyl group and the terminal NH_2_; and between the L‐tyrosine's carbonyl group and the D‐tryptophan's amide group. Compared to the conformer aq‐I, it seems that the stabilizing interactions, which involve two distinct parts of the molecule, cause the aq‐II to form a more distorted structure that is closer to a common helix. Indeed, the commonly used *φ* and *ψ* dihedral angle values that describe the orientation of a peptide's backbone are different from those of the typical α‐helix (Table 4). The backbone orientations of all the conformers are schematically depicted in Supplementary Information, Figure S4.

**TABLE 4:** *φ* and *ψ* angles of the two stable conformers in aqueous solution (designated aq.) and three stable conformers in the presence of SDS micelles (designated mic.) defining orientation of the GHRP‐5’s backbone. The numbering of the angles starts from the tyrosine end.

Conformer	*φ*_1_ (°)	*ψ*_1_ (°)	*φ*_2_ (°)	*ψ*_2_ (°)	*φ*_3_ (°)	*ψ*_3_ (°)	*φ*_4_ (°)	*ψ*_4_ (°)
aq‐I	46.2	−131.3	−95.9	19.6	125.8	41.5	−91.0	76.7
aq‐II	108.5	−148.6	−80.4	75.8	77.5	−74.8	−114.4	−178.0
mic‐I	78.5	−72.8	−66.8	94.2	48.2	−146.1	−135.6	105.3
mic‐II	45.9	−132.6	−101.2	30.0	112.7	44.3	−109.5	94.2
mic‐III	77.8	−70.4	−65.7	92.9	47.0	−140.6	−167.8	161.7

**FIGURE 7:**
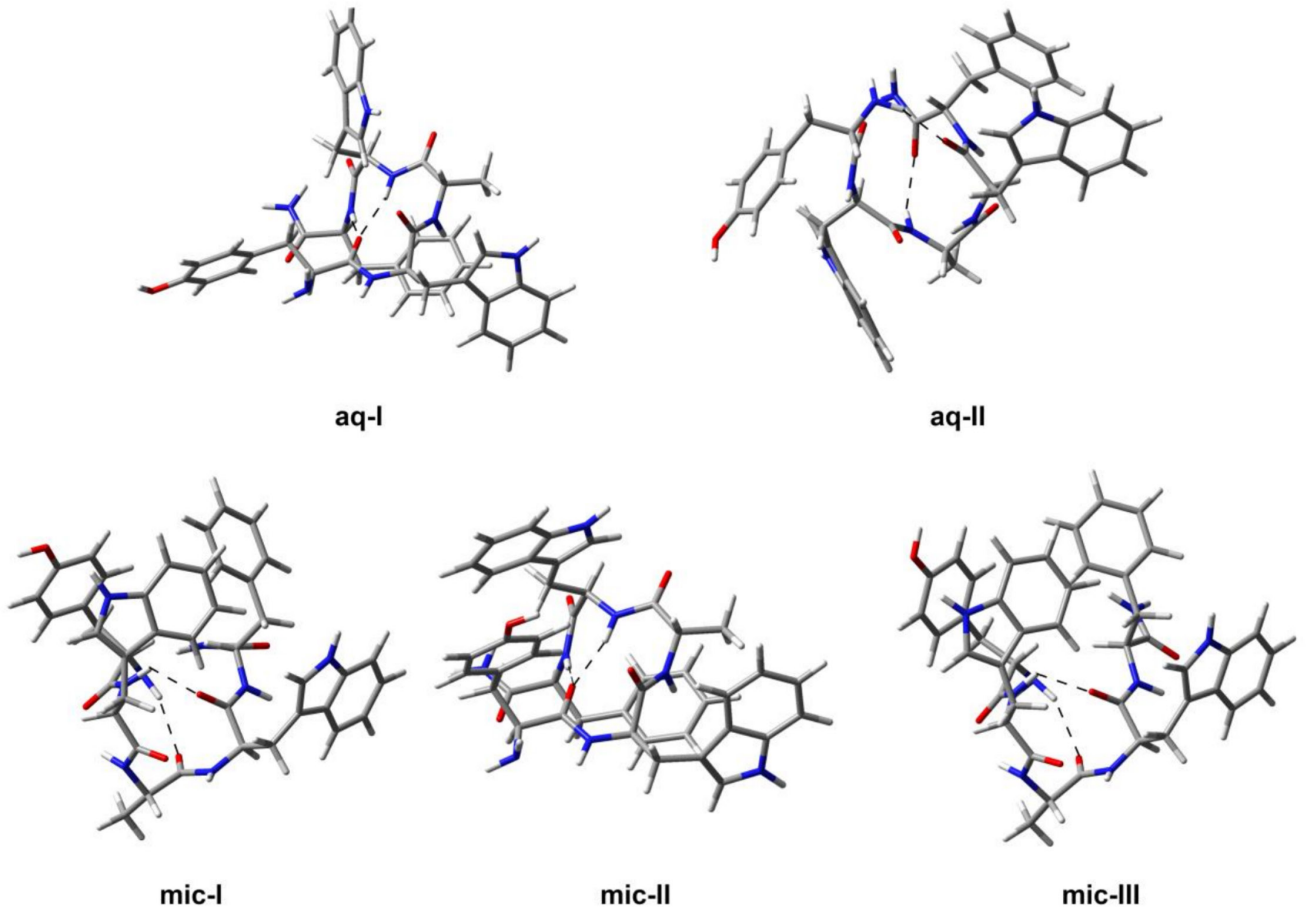
The structures of two stable conformers of GHRP‐5 in aqueous solution and three stable conformers in the presence of SDS micelles that exhibit similar ECD spectra to those observed experimentally. Stabilizing hydrogen bonds are highlighted with dashed lines.

After a closer look at the conformers' structures that are considered stable in the presence of SDS micelles (Figure 7), it was revealed that two of them (conformers mic‐I and mic‐III) share very similar orientation of the backbone given by the *φ* and *ψ* dihedral angles (Table 4), which differ significantly only for the *φ*_4_ and *ψ*_4_ angles between the L‐tryptophan and D‐phenylalanine residues. Both structures are stabilized by two C=O … H‐N interactions between L‐tyrosine and D‐phenylalanine and between L‐alanine and terminal NH_2_. On the other hand, the structure of conformer mic‐II is stabilized by the interaction of L‐tyrosine's carbonyl group with both the L‐tryptophan and L‐alanine amide groups in the same way as in the case of conformer aq‐I. Their *φ* and *ψ* angle values, and consequently the orientation of the backbones in both structures, are very close as well (see Supplementary Information, Figure S11).

After a visual comparison of the conformers predicted stable for aqueous solutions and those predicted to be predominant in the presence of micelles, it is clear that the mic‐I and mic‐III conformers prefer backbone orientations that more resemble a helical structure, especially if compared to the aq‐I conformer. Although the *φ* and *ψ* angles do not have values typical for those found in naturally occurring α‐helices, it is not surprising as the studied GHRP is composed only of five amino acids and, thus, a resulting helix cannot be stabilized in the same way as in the case of longer peptides and proteins. Furthermore, it is composed of both D‐ and L‐amino acids, which is also different from naturally occurring peptides that form typical α‐helices. The experimental ECD spectrum of GHRP‐5 with SDS micelles is also not typical for an α‐helix—the negative bands are much better resolved and the positive band at 231 nm is present. It can thus be concluded that SDS micelles probably cause GHRP‐5 to prefer the described unusual types of helical structures, which we consider to be a particularly interesting phenomenon.

## 4 Conclusions

In this work, we present and interpret the ECD spectra of eight different GHRPs. Emphasis was placed mainly on the description of individual differences in spectra with respect to the differences in the GHRP structures. Two pairs of the studied compounds share very similar structural moieties, namely GHRP‐1 with GHRP‐2, and GHRP‐5 with GHRP‐6. The UV and ECD spectra of both respective pairs exhibited similar spectral patterns, but certain differences were also recognized, which showed that the different substituents can play an important role in the preferred secondary structures. We also tested the thermal stability of the studied compounds. We used SDS micelles as a simple membrane model and we analyzed how their presence affects the secondary structure of the GHRPs. In the case of GHRP‐2, GHRP‐3, and GHRP‐4, particle aggregation was observed, which did not allow an ECD analysis of the solutions. In the case of anamorelin, the band positions and relative intensities in the ECD spectrum did not change; the absolute intensity, however, increased in the solution containing SDS micelles. On the other hand, the ECD spectrum of GHRP‐1 changed with SDS micelles, indicating that an interaction took place. The most interesting changes were observed for the GHRP‐5 and SDS micelles system, where an ECD signal indicating the formation of a helical secondary structure was induced. In order to interpret the observed changes, a conformational search was performed and the ECD spectra were calculated for the 20 lowest‐energy conformers, and those that contribute to the observed ECD spectra with an increased probability were identified. It turned out that GHRP‐5 can indeed form a helical structure stabilized by the intramolecular hydrogen bonds, and the calculated ECD spectra were in solid agreement with the experimental features.

We demonstrated that ECD spectroscopy is a suitable tool for studying secondary structures and interactions of oligopeptides with biological importance. Combined with theoretical calculations, the achieved results indicate distinct conformational changes in GHRP‐5’s secondary structure induced by the SDS micelles. Such knowledge can possibly bring further insight into the mechanism of action and biological activity of this important class of compounds. For the future, other membrane models such as liposomes can be used for interaction studies.

## Funding

This work was supported by the Ministry of Interior of the Czech Republic (VJ01010043).

## Supporting information


**Figure S1:** Chemical structures of the studied GHRPs with marked absolute configurations of the stereogenic centers.
**Figure S2:** Stable conformers of the selected GHRPs with a similar arrangement of the indole and phenyl rings.
**Figure S3:** The ECD (top) and UV absorption spectra (bottom) of GHRP‐2 under different temperatures.
**Figure S4:** The ECD (top) and UV absorption spectra (bottom) of GHRP‐3 under different temperatures.
**Figure S5:** The ECD (top) and UV absorption spectra (bottom) of GHRP‐5 under different temperatures.
**Figure S6:** The ECD (top) and UV absorption spectra (bottom) of GHRP‐6 under different temperatures.
**Figure S7:** The ECD (top) and UV absorption spectra (bottom) of ipamorelin under different temperatures.
**Figure S8:** The ECD (top) and UV absorption spectra (bottom) of GHRP‐5 in the presence of SDS micelles under different temperatures.
**Figure S9:** Comparison of the ECD (top) and UV absorption spectra (bottom) of GHRP‐5 in the presence of SDS micelles measured after a period of 5 days.
**Figure S10:** The calculated ECD spectra (CAM‐B3LYP/6‐31+G(d,p)/PCM) of two conformers found to be predominant in an aqueous solution (top left), three conformers found to be predominant in the presence of SDS micelles (top right), and the remaining 15 conformers (bottom), which were divided into two parts for better clarity.
**Figure S11:** Schematic backbone orientations of the two stable conformers predicted for aqueous solution (aq., top row) and three stable conformers predicted in the presence of micelles (mic., bottom row).

## Data Availability

The data that support the findings of this study are available from the corresponding author upon reasonable request.
