# Supplementary material for: Growth Hormone–Releasing Peptides: Investigation of Their Secondary Structure, Thermal Stability, and Model Membrane Interactions
Source: Chirality. 2026 Jan 19;38(2):e70083. doi: 10.1002/chir.70083 (PMC12869127; doi:10.1002/chir.70083)
Supplement: Supplementary file 1 — Figure S1: Chemical structures of the studied GHRPs with marked absolute configurations of the stereogenic centers. Figure S2: Stable conformers of the selected GHRPs with a similar arrangement of the indole and phenyl rings. Figure S3: The ECD (top) and UV absorption spectra (bottom) of GHRP‐2 under different temperatures. Figure S4: The ECD (top) and UV absorption spectra (bottom) of GHRP‐3 under different temperatures. Figure S5: The ECD (top) and UV absorption spectra (bottom) of GHRP‐5 under different temperatures. Figure S6: The ECD (top) and UV absorption spectra (bottom) of GHRP‐6 under different temperatures. Figure S7: The ECD (top) and UV absorption spectra (bottom) of ipamorelin under different temperatures. Figure S8: The ECD (top) and UV absorption spectra (bottom) of GHRP‐5 in the presence of SDS micelles under different temperatures. Figure S9: Comparison of the ECD (top) and UV absorption spectra (bottom) of GHRP‐5 in the presence of SDS micelles measured after a period of 5 days. Figure S10: The calculated ECD spectra (CAM‐B3LYP/6‐31+G(d,p)/PCM) of two conformers found to be predominant in an aqueous solution (top left), three conformers found to be predominant in the presence of SDS micelles (top right), and the remaining 15 conformers (bottom), which were divided into two parts for better clarity. Figure S11: Schematic backbone orientations of the two stable conformers predicted for aqueous solution (aq., top row) and three stable conformers predicted in the presence of micelles (mic., bottom row). [file CHIR-38-e70083-s001.docx]

SUPPLEMENTARY MATERIAL

Growth Hormone-Releasing Peptides: Investigation of Their Secondary Structure, Thermal Stability, and Model Membrane Interactions

Králík F.^a^, Kvíčalová A. ^a^, Salaďáková A. ^a^, Kuchař M.^b^, Setnička V.^a^

^a^ Department of Analytical Chemistry, University of Chemistry and Technology, Technická 5, Prague 6, 166 28, Czech Republic

^b^ Forensic Laboratory of Biologically Active Substances and Department of Chemistry of Natural Compounds, University of Chemistry and Technology, Technická 5, Prague 6, 166 28, Czech Republic

Corresponding authors: [Frantisek.Kralik@vscht.cz](mailto:Frantisek.Kralik@vscht.cz), [Vladimir.Setnicka@vscht.cz](mailto:Vladimir.Setnicka@vscht.cz)

Contents

[1. Chemical structures of the studied GHRPs 2](#_Toc216880777)

[2. Stable conformers of GHRP-1, GHRP-2, GHRP-5 and GHRP-6 with a similar arrangement of the indole and phenyl rings 4](#_Toc216880778)

[3. Temperature stability of the studied GHRPs 5](#_Toc216880779)

[4. Temperature stability of GHRP-5 in the presence of SDS micelles 10](#_Toc216880780)

[5. Time stability of GHRP-5 in the presence of SDS micelles 11](#_Toc216880781)

[6. DFT-calculated spectra of the 20 lowest-energy conformers of GHRP-5 12](#_Toc216880782)

[7. Schematic backbone orientations of the stable conformers of GHRP-5 13](#_Toc216880783)

[8. Cartesian coordinates of stable conformers of GHRP-5 14](#_Toc216880784)

# Chemical structures of the studied GHRPs


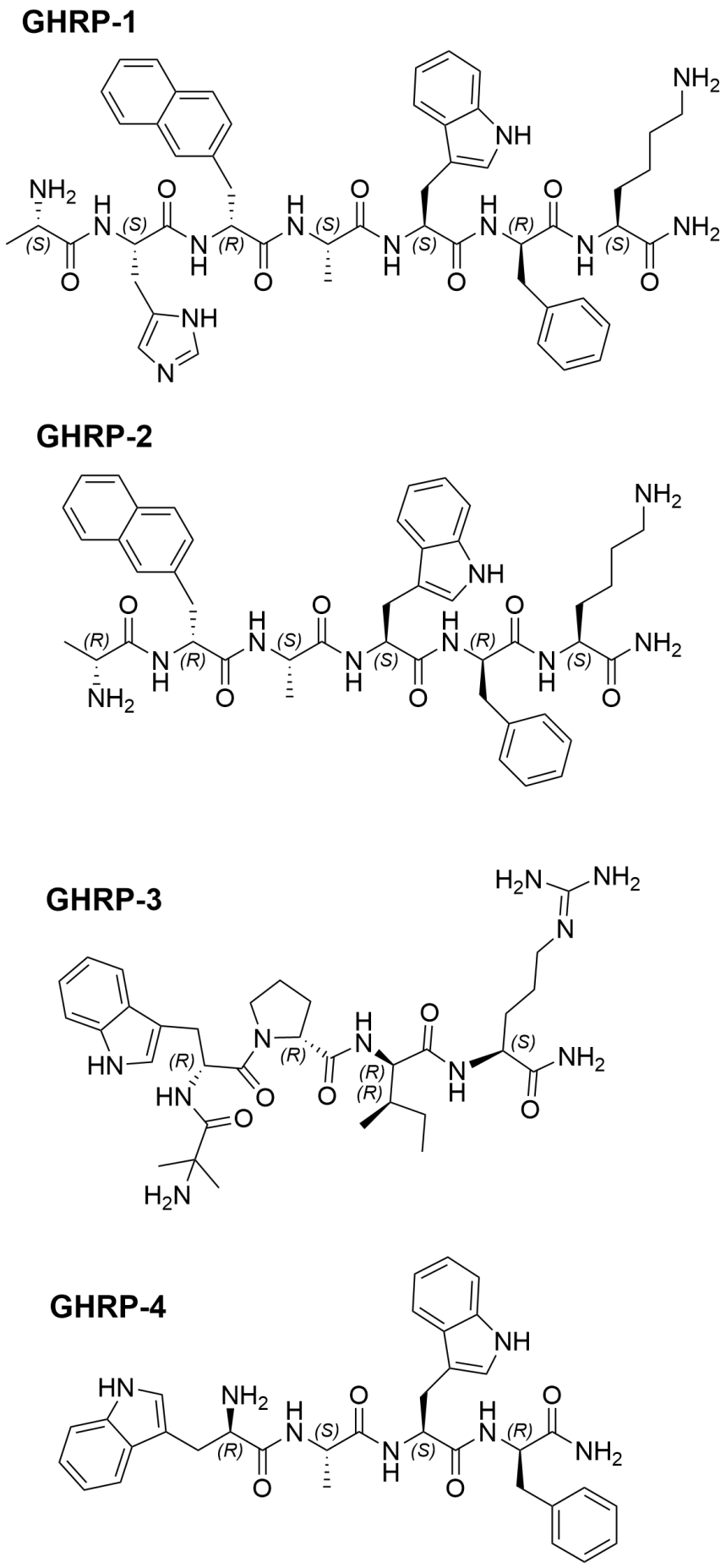


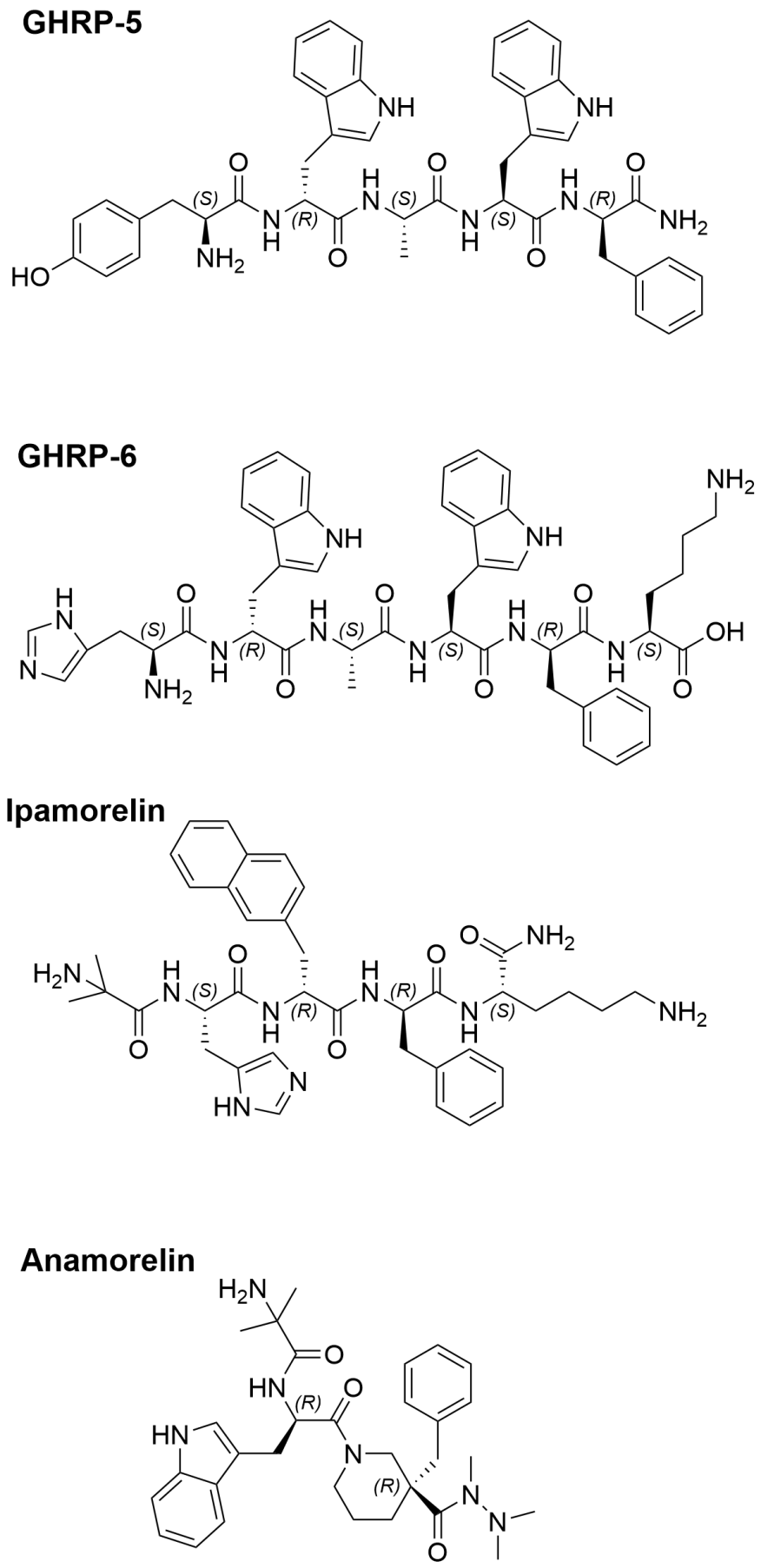


**Fig. S1** Chemical structures of the studied GHRPs with marked absolute configurations of the stereogenic centres.

# Stable conformers of GHRP-1, GHRP-2, GHRP-5 and GHRP-6 with a similar arrangement of the indole and phenyl rings


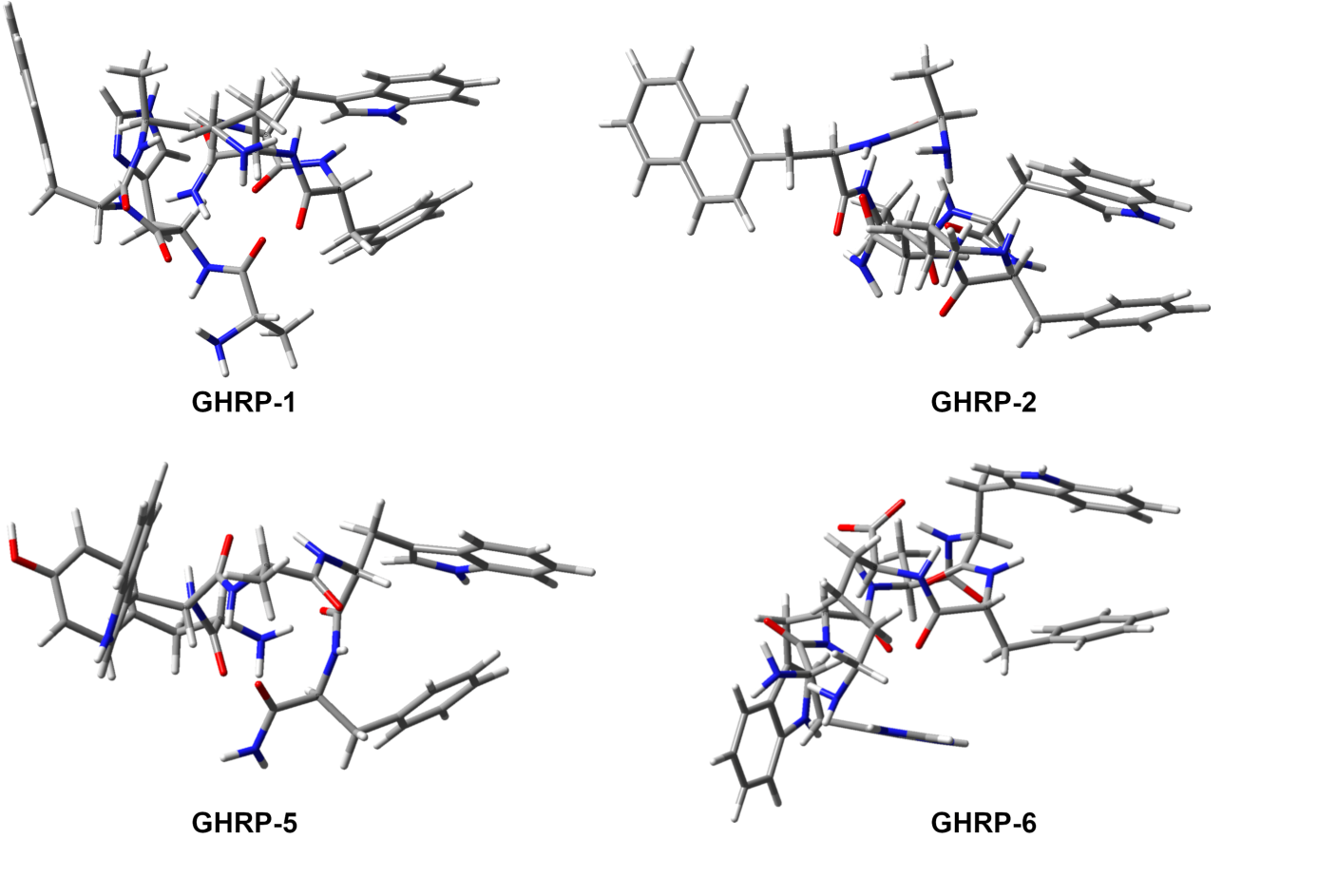


**Fig. S2** Stable conformers of the selected GHRPs with a similar arrangement of the indole and phenyl rings.

# Temperature stability of the studied GHRPs


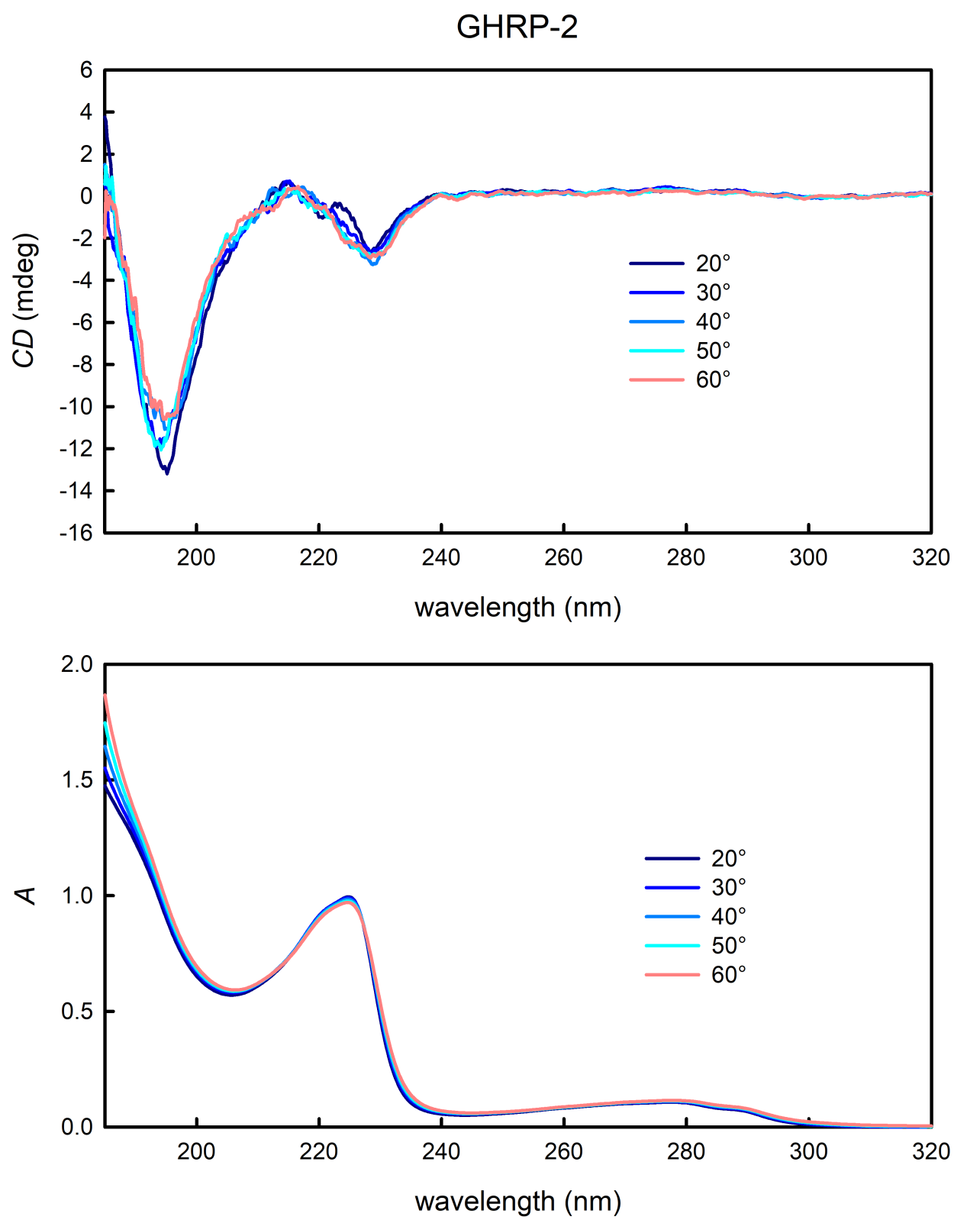


**Fig. S3** The ECD (top) and UV absorption spectra (bottom) of GHRP-2 under different temperatures.


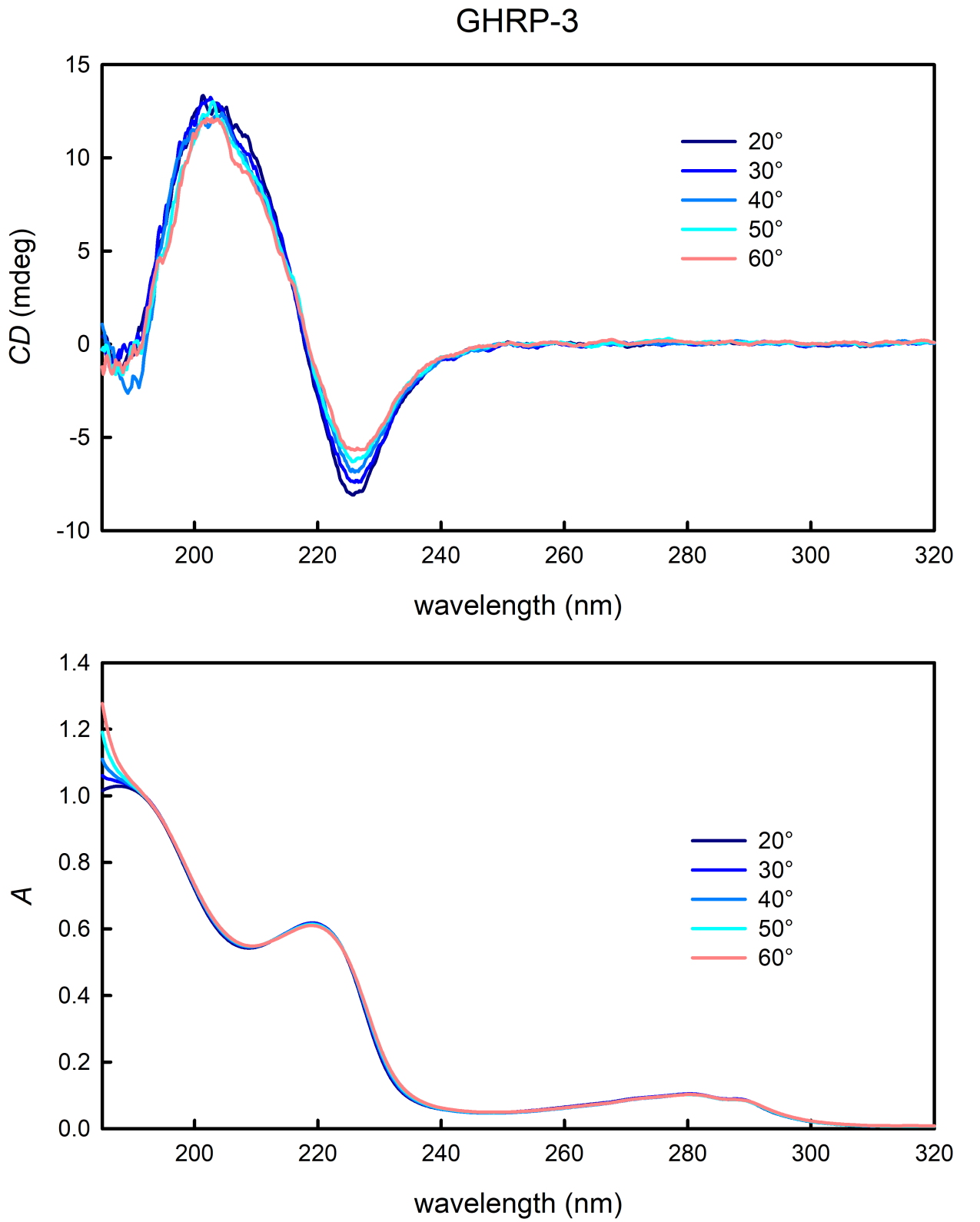


**Fig. S4** The ECD (top) and UV absorption spectra (bottom) of GHRP-3 under different temperatures.


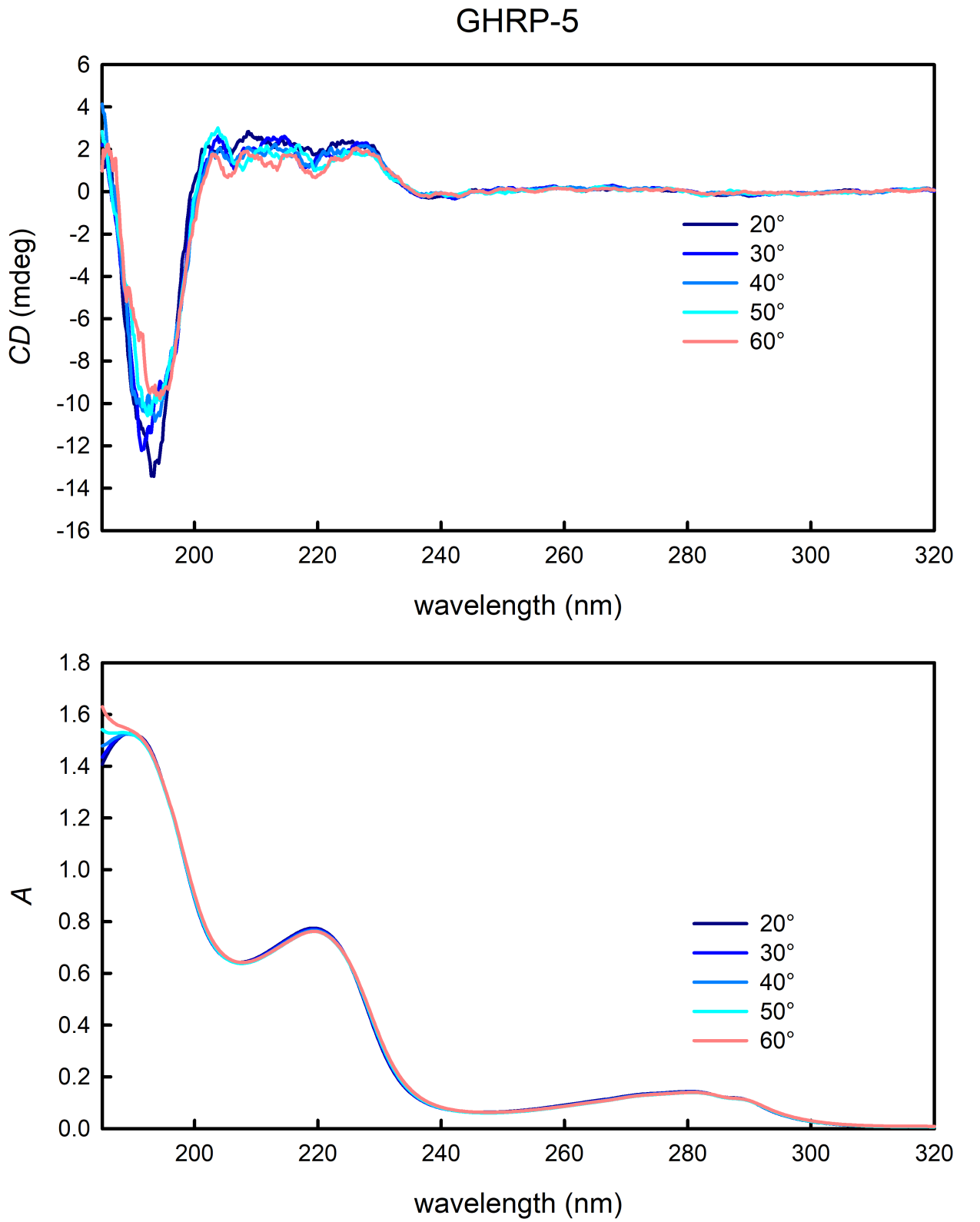


**Fig. S5** The ECD (top) and UV absorption spectra (bottom) of GHRP-5 under different temperatures.


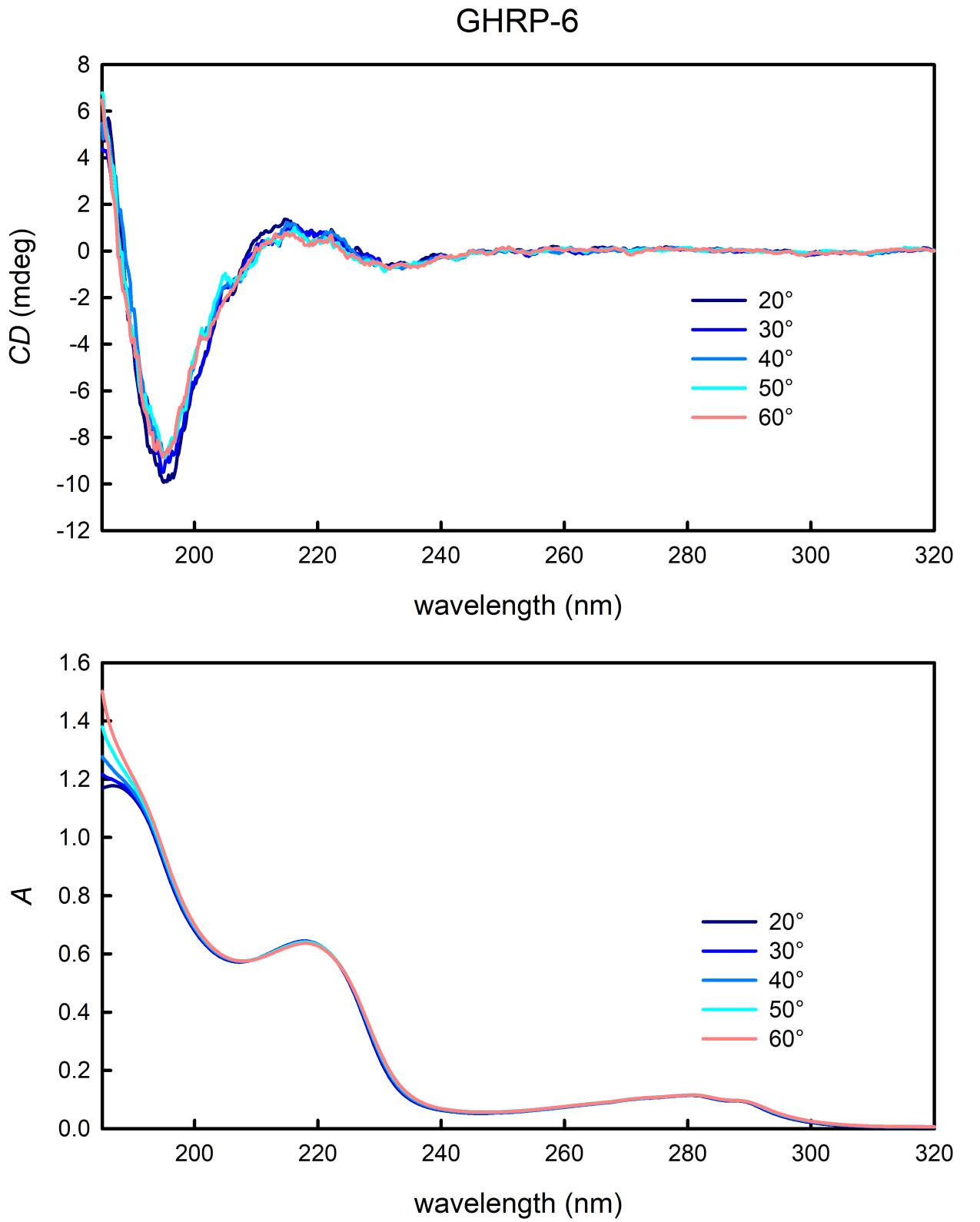


**Fig. S6** The ECD (top) and UV absorption spectra (bottom) of GHRP-6 under different temperatures.


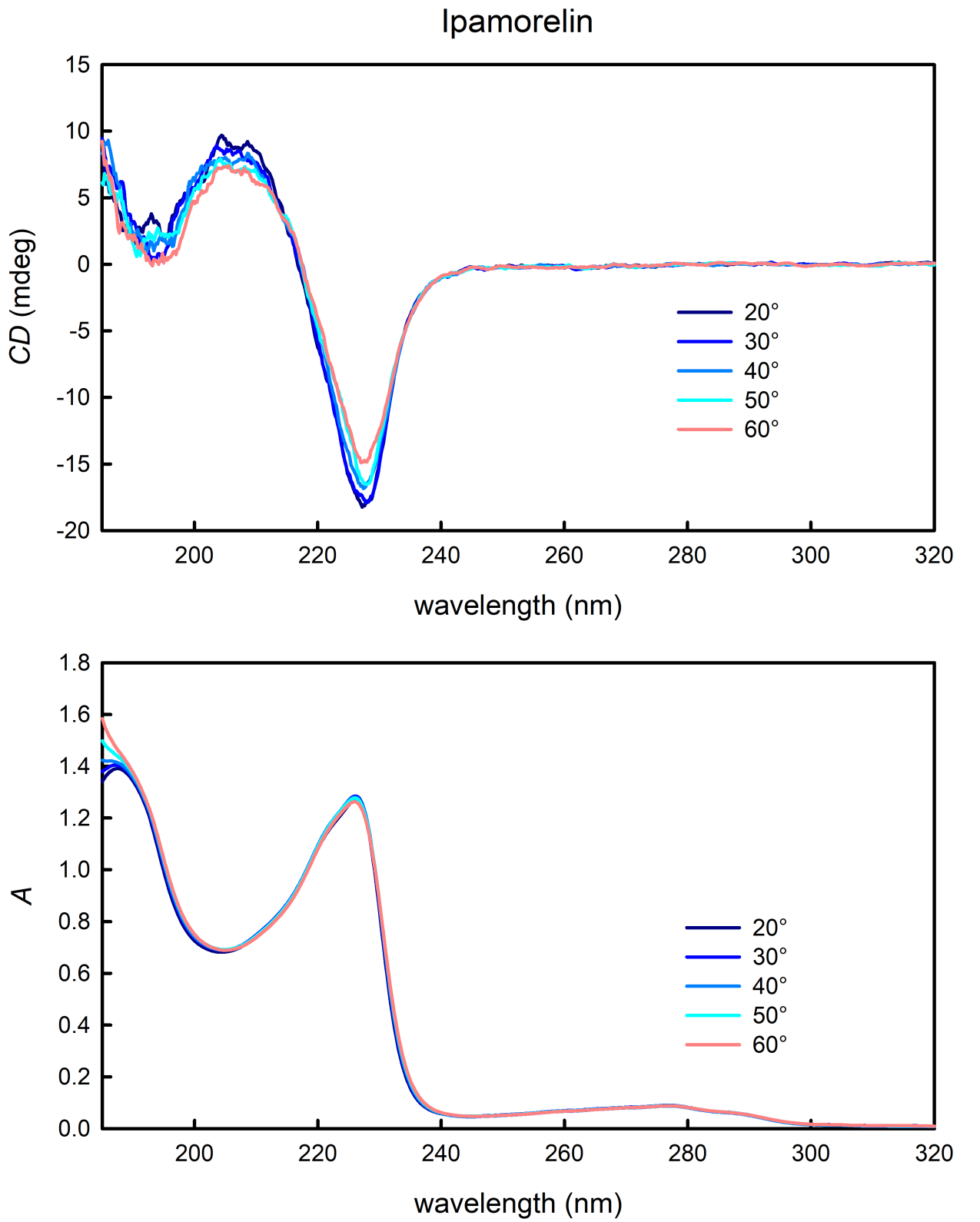


**Fig. S7** The ECD (top) and UV absorption spectra (bottom) of ipamorelin under different temperatures.

# Temperature stability of GHRP-5 in the presence of SDS micelles


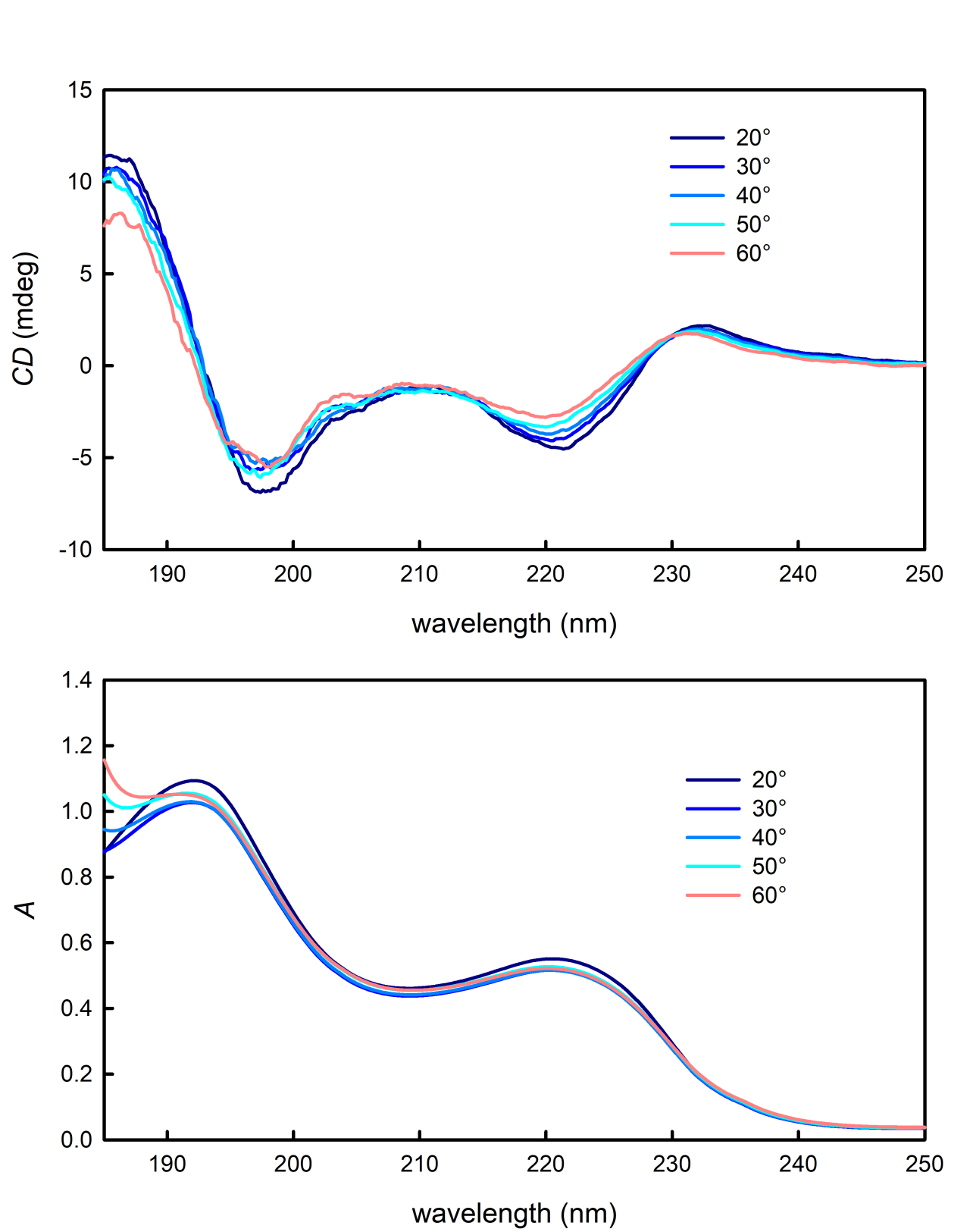


**Fig. S8** The ECD (top) and UV absorption spectra (bottom) of GHRP-5 in the presence of SDS micelles under different temperatures.

# Time stability of GHRP-5 in the presence of SDS micelles


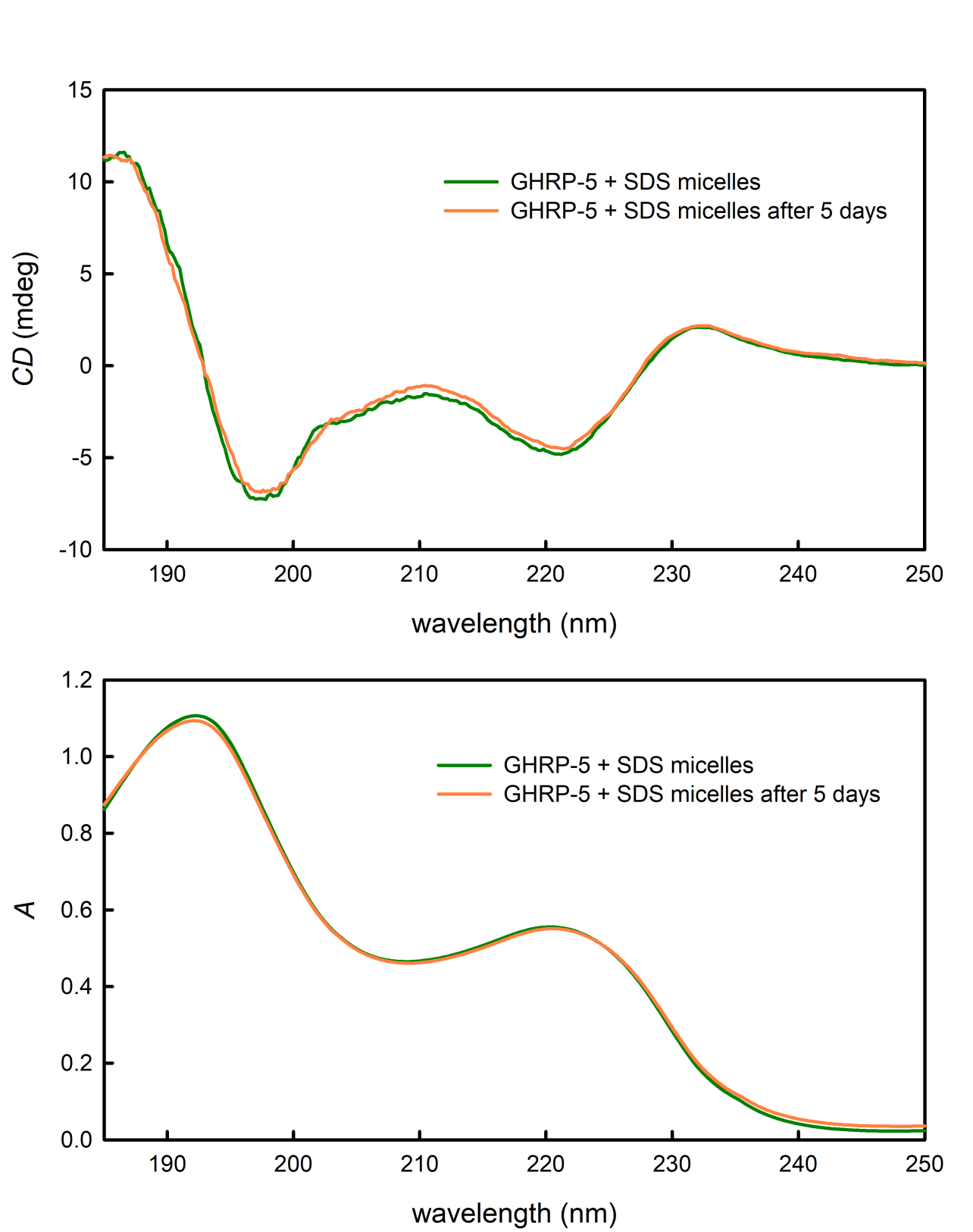


**Fig. S9** Comparison of the ECD (top) and UV absorption spectra (bottom) of GHRP-5 in the presence of SDS micelles measured after a period of 5 days.

# DFT-calculated spectra of the 20 lowest-energy conformers of GHRP-5


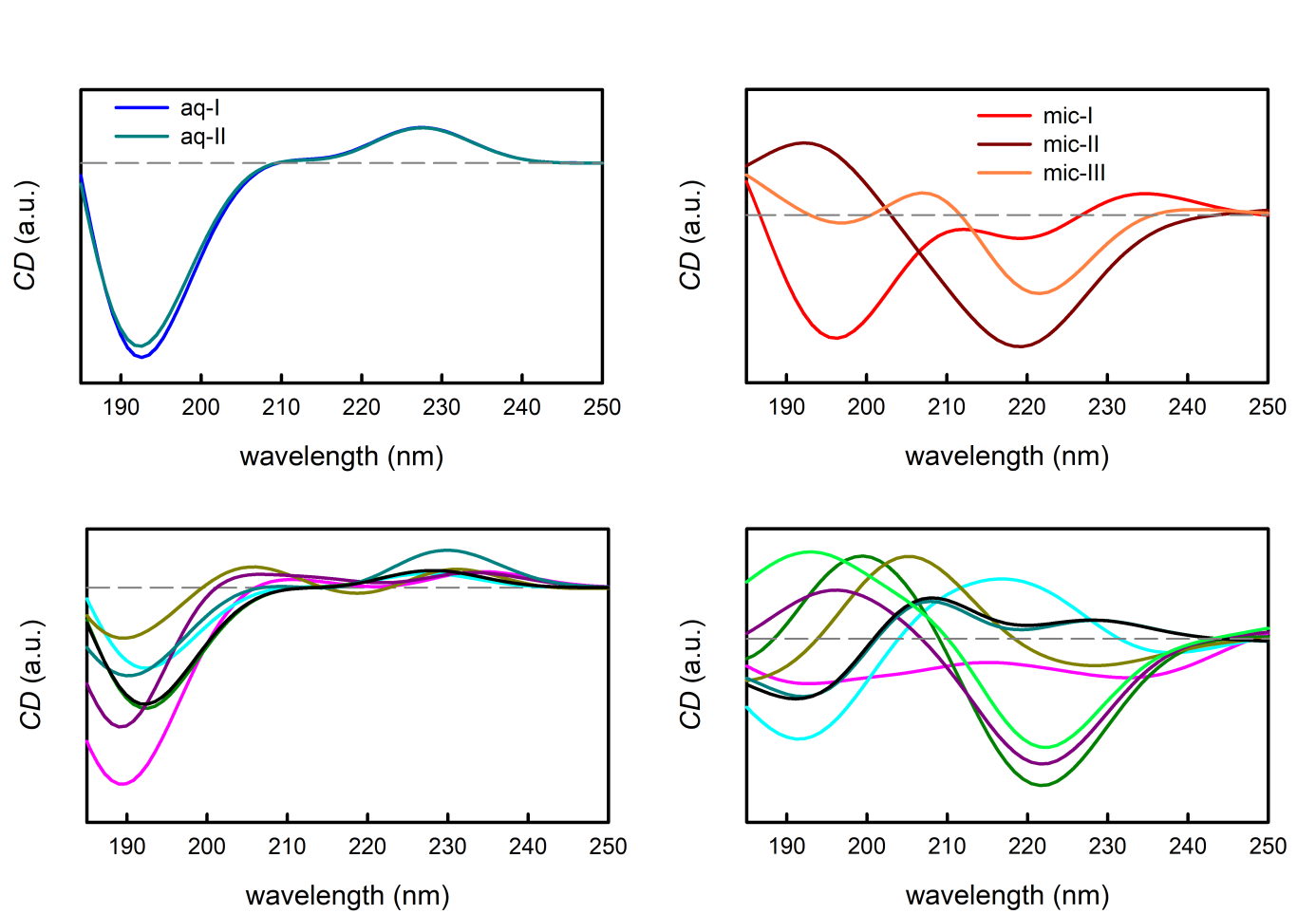


**Fig. S10** The calculated ECD spectra (CAM-B3LYP/6-31+G(d,p)/PCM) of two conformers found to be predominant in an aqueous solution (top left), three conformers found to be predominant in the presence of SDS micelles (top right), and the remaining 15 conformers (bottom), which were divided into two parts for better clarity.

# Schematic backbone orientations of the stable conformers of GHRP-5


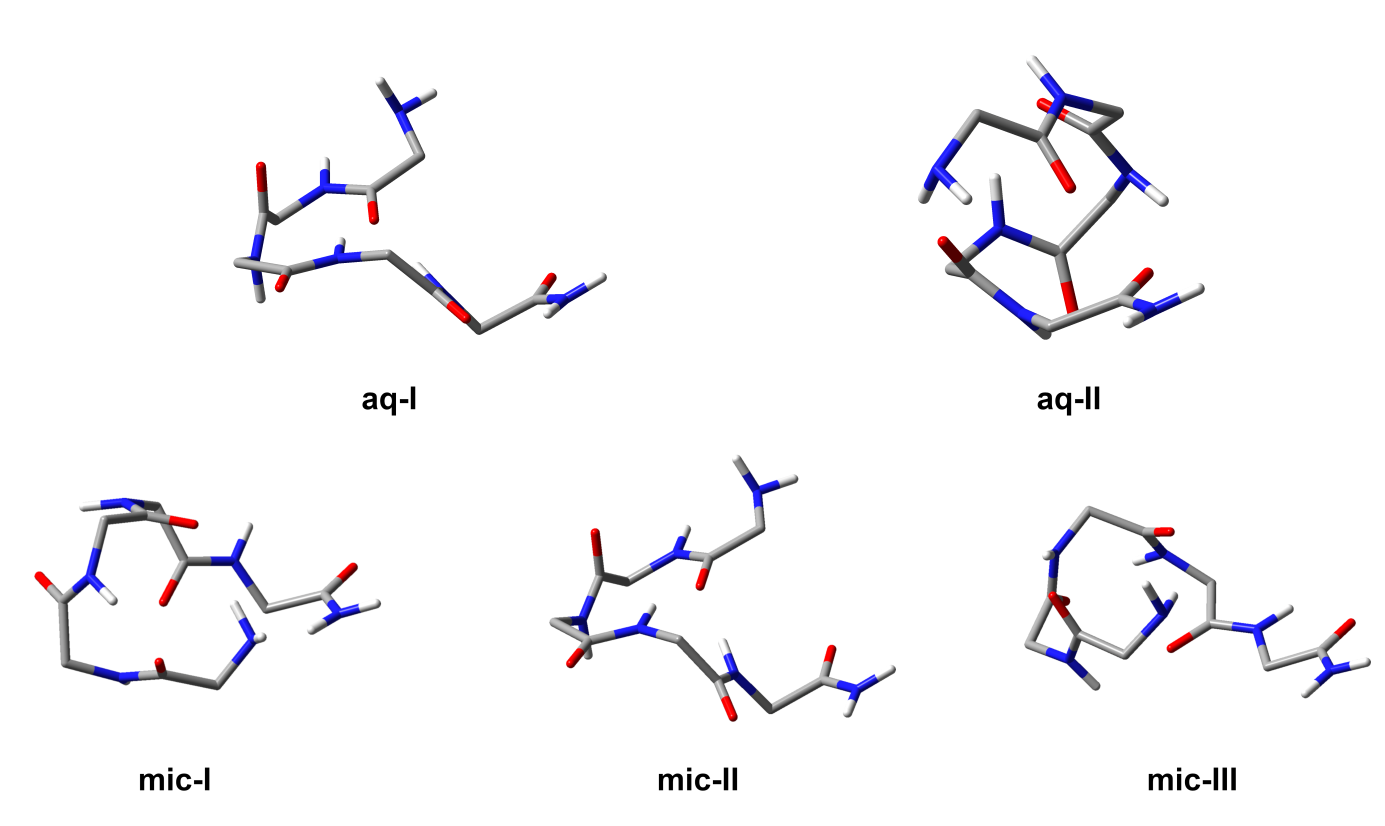


**Fig. S11** Schematic backbone orientations of the two stable conformers predicted for aqueous solution (aq., top row) and three stable conformers predicted in the presence of micelles (mic., bottom row).

# Cartesian coordinates of stable conformers of GHRP-5

For each conformer, the Cartesian coordinates are given in the following order: atom type, x-, y-, z-coordinate.

*Conformer aq-I*

O 0.3377957298 -0.3907856631 -2.6084454389

O -3.1533433597 -0.1761242166 3.2312439196

O -3.1532531286 -2.7148504802 -0.5372474858

O 0.6223434500 0.5996155357 0.2530954391

O 0.3766188261 2.4881445165 4.2908199292

O 1.7053028677 8.6636656376 2.1409197398

N -1.7779685287 -1.0356191311 0.1025716097

N 2.4817344595 0.6150325196 -1.0023347955

N 0.3575218434 -2.1409454040 -1.1805900744

N -1.0569642520 0.1887705565 2.4975729599

N -3.6413565094 0.5754375040 -3.3932379194

N 2.5654425248 -4.6585343936 -3.4583359624

N 2.7420904698 3.2273040247 -0.9203177576

N -1.7861848294 2.0220375504 4.7404796356

C -2.7578183734 -0.2825995784 0.8510620245

C 2.3582938306 -0.7846064568 -1.3528941112

C -2.9618619107 1.0996667741 0.1928246970

C 3.2702181443 -1.1553689435 -2.5308222495

C -3.5063317664 0.9368685540 -1.1856224106

C 3.2108498871 -2.6315228419 -2.7511806077

C -0.9545605306 -2.6411679642 -1.5222381924

C 0.9096617525 -1.0689922402 -1.7633188071

C -4.8859018895 0.8851398940 -1.5502874776

C 3.9547411898 -3.6177187569 -2.0341060615

C -2.3440432619 -0.0961389934 2.3066839156

C -2.0748715757 -2.1365608167 -0.5933568782

C -4.9274753455 0.6597901282 -2.9487742214

C -0.4525940584 0.2516367835 3.8154482159

C 3.5181915504 -4.8794803069 -2.5081096224

C -2.7932965907 0.7320280451 -2.3331764567

C 2.3829165524 -3.3117762305 -3.5976333503

C 1.8794036017 2.6670030506 0.1060243155

C 1.5928369910 1.2050521838 -0.2154031511

C -0.9396948444 -4.1638384469 -1.5718373113

C 1.0009498120 -0.2094543054 3.7446703323

C -6.0799692672 1.0056148223 -0.8400587710

C 4.9513177334 -3.5595037969 -1.0589648205

C 0.5552545989 3.4299504485 0.3391561368

C -6.1311682877 0.5616672271 -3.6359510694

C 4.0435038328 -6.0669118415 -2.0119386462

C 1.2209904441 -1.4652262829 2.9435200050

C -0.5630331271 1.7052969722 4.3146309427

C -7.2683952421 0.9048730658 -1.5272431637

C 5.4663688113 -4.7395200727 -0.5718176016

C -7.2946373439 0.6871325806 -2.9081928045

C 5.0149079581 -5.9782408200 -1.0385006631

C 0.8243090583 4.8314737891 0.8027032573

C 0.2448985119 -2.4438870203 2.7987229148

C 2.4570919289 -1.6682948527 2.3385470721

C 0.8643579086 5.8885296483 -0.0957765336

C 1.0873226387 5.0872783685 2.1452283424

C 0.4869945134 -3.5782161629 2.0415407493

C 2.7089019809 -2.8092613481 1.5977821067

C 1.1553838226 7.1739271101 0.3262870749

C 1.3783089727 6.3650810822 2.5764104986

C 1.7165288757 -3.7633233975 1.4304913244

C 1.4155726412 7.4205566235 1.6681225606

H -3.6958916298 -0.8482459957 0.8314000390

H 2.6108855451 -1.3949686399 -0.4773136810

H -1.9954975818 1.6064199012 0.1593797262

H -3.6506761342 1.6829163543 0.8046640511

H 2.9277565334 -0.6247912003 -3.4206338322

H 4.2919773541 -0.8532121025 -2.3001496517

H -1.2012059077 -2.2334055746 -2.5119524566

H -0.8917148231 -0.5730833755 -0.0660252241

H 3.2253399030 1.1878386295 -1.3835736190

H 0.8486552795 -2.6257983412 -0.4400284872

H -1.0481765720 -0.3805933731 4.4834420760

H -1.7294384481 0.6802874797 -2.4714877516

H 1.6705251267 -2.9188696712 -4.2973229784

H -0.4320783626 0.3297857128 1.7092040067

H 2.4386882062 2.6695338166 1.0516559256

H -3.3572248549 0.4076461895 -4.3429967729

H 2.0932019334 -5.3701969848 -3.9894595260

H -0.2623861853 -4.5079616137 -2.3467368638

H -0.6202474420 -4.5618580951 -0.6124937059

H -1.9446155178 -4.5213722132 -1.7759748261

H 1.3627432685 -0.3584354285 4.7654078662

H 1.5906449574 0.6033869884 3.3126098274

H -6.0698979352 1.1673677878 0.2280628475

H 5.3176566995 -2.6078612326 -0.7030406814

H -0.0191527668 3.4329564812 -0.5910019208

H -0.0147789523 2.8921258707 1.0985186148

H -6.1537177844 0.3897727436 -4.7014035575

H 3.7045374102 -7.0241222119 -2.3778138624

H -8.2039254593 0.9924762113 -0.9942337449

H 6.2365907793 -4.7173205553 0.1853625270

H -8.2482915884 0.6138462343 -3.4094069662

H 5.4440075141 -6.8806812636 -0.6292708292

H 2.2087582897 3.3581742999 -1.7749251245

H 3.0685693910 4.1414527844 -0.6275277963

H -0.7180032167 -2.3338382585 3.2728316191

H 3.2298970409 -0.9229420614 2.4569972196

H 0.6540401220 5.7139762507 -1.1412603564

H 1.0516854349 4.2799778822 2.8629319465

H -1.9878953097 2.9709290022 5.0152164334

H -2.5453524913 1.3634615377 4.6279264002

H -0.2926047996 -4.3187882723 1.9375120658

H 3.6777498300 -2.9655120014 1.1478398468

H 1.1758606515 7.9835649220 -0.3888226649

H 1.5768540424 6.5692741162 3.6166483858

H 1.9145507537 -4.6485919664 0.8440674769

H 1.6885194456 9.3145868658 1.4231791035

*Conformer aq-II*

O 1.2613778952 -0.7801217608 -1.9974616909

O -1.3308208067 1.6159435772 -0.0282805005

O -2.0962018133 -2.8301406892 0.0426545526

O 1.5138624175 1.4581982053 1.8044772450

O 0.2931918576 -1.4976659871 2.2603762096

O 7.5854189908 1.2178921012 -1.3868304123

N -1.4708322819 -1.2970854132 -1.4973802142

N 2.4069968284 0.9131645702 -0.1992867689

N 1.0300812504 -2.3644260888 -0.4012530328

N -1.8241929472 -0.1940198631 1.2301249398

N -5.0501121533 3.3927074547 -1.3183449660

N 4.4632939143 -4.4072558495 1.0228200062

N 0.6052497422 3.9095842785 0.7240813112

N 0.4575265917 -0.1794094624 4.0758948908

C -2.4291207952 -0.2824050961 -1.0886865463

C 2.5217163419 -0.5094888935 0.0304846835

C -2.7709447510 0.6448538976 -2.2539634702

C 3.9529162923 -0.9942057726 -0.2804622397

C -3.8941564001 1.5598682223 -1.8911663680

C 4.1011696100 -2.4547602742 -0.0196024482

C 0.0216570902 -3.1427210747 -1.1094689299

C 1.5321833078 -1.2264091679 -0.8808154246

C -5.2722741479 1.2043101617 -1.7697523649

C 3.8274598003 -3.5147039557 -0.9378165958

C -1.8098799123 0.4944719149 0.0867243424

C -1.3047412098 -2.4345229650 -0.8060695089

C -5.9655494451 2.3875935538 -1.4105809249

C -1.3033330928 0.2998424364 2.4825162218

C 4.0680508710 -4.7260671901 -0.2422170454

C -3.8116423850 2.8897378598 -1.5976051041

C 4.4795017043 -3.0482614246 1.1515053274

C 1.7586158252 3.2189942537 0.1732216350

C 1.8621001219 1.7745729369 0.6699439675

C 0.0393402935 -4.5794954771 -0.6227162638

C -2.4322827818 0.2807837380 3.5326973095

C -5.9855949415 0.0161651870 -1.9308209237

C 3.4103748902 -3.5611571488 -2.2685449318

C 3.0493977947 3.9625078273 0.5800155030

C -7.3421567244 2.3973362640 -1.2208854341

C 3.8928649023 -5.9642669215 -0.8496498562

C -3.5941013496 1.0832548093 3.0149339482

C -0.0940503865 -0.5352120782 2.9144544820

C -7.3479793566 0.0320708846 -1.7322714601

C 3.2360551169 -4.7905769122 -2.8623022969

C -8.0193399698 1.2080119003 -1.3828457170

C 3.4738299138 -5.9781583869 -2.1623168192

C 4.2728512791 3.2707310362 0.0549908133

C -3.6443164289 2.4566860716 3.2124969189

C -4.6006273830 0.4743370068 2.2756094827

C 5.0614375033 2.4859430243 0.8875399107

C 4.6212155985 3.3657574402 -1.2862766119

C -4.6847526468 3.2050962887 2.6914333777

C -5.6409235292 1.2204341284 1.7520922631

C 6.1633393180 1.8121739842 0.3994846845

C 5.7199105859 2.6933978098 -1.7889920647

C -5.6853217253 2.5879435238 1.9609479004

C 6.4989759810 1.9069472544 -0.9481539551

H -3.3268149356 -0.7965298278 -0.7289915613

H 2.2662791484 -0.6981341309 1.0759889605

H -3.0473694254 0.0334825982 -3.1148974327

H -1.8853164584 1.2335333556 -2.5007916225

H 4.1652670520 -0.7770290121 -1.3294677467

H 4.6455461238 -0.4205249624 0.3371137512

H 0.2462674546 -3.0761393800 -2.1783840093

H -0.6977394421 -0.9762953387 -2.0748281414

H 2.5716989226 1.2097830112 -1.1504702668

H 1.1214685894 -2.5564407672 0.5880098188

H -0.9606384156 1.3246013562 2.3009353409

H -2.9392337054 3.5126876135 -1.5688461279

H 4.7700264187 -2.5852984952 2.0740789526

H -2.1403419816 -1.1575533843 1.2180876722

H 1.6614966634 3.2166904184 -0.9165981893

H -5.2453608536 4.3444490110 -1.0595984766

H 4.7099968140 -5.0647991267 1.7427914560

H -0.2447568655 -4.6096925687 0.4261198212

H -0.6754771875 -5.1668381519 -1.1904486754

H 1.0329636991 -4.9995104770 -0.7482885434

H -2.0700221686 0.6996973364 4.4696419857

H -2.7318688770 -0.7550605589 3.6988699366

H -5.4782267175 -0.8972527335 -2.2052735764

H 3.2181740185 -2.6500123614 -2.8168493684

H 2.9784354236 4.9788968357 0.1925782304

H 3.0833470979 4.0085509282 1.6697554856

H -7.8631723694 3.3046479182 -0.9548403150

H 4.0777075645 -6.8815875723 -0.3123276169

H -7.9164793252 -0.8792280949 -1.8486876533

H 2.9091827264 -4.8467190989 -3.8902801307

H -9.0891479820 1.1781162390 -1.2387860423

H 3.3247644282 -6.9214762376 -2.6655873773

H -0.2374528279 3.4214942591 0.4293465984

H 0.6444767390 3.8324062392 1.7376413383

H -2.8648463490 2.9442357501 3.7794329812

H -4.5731387500 -0.5933203851 2.1110671024

H 4.8058231227 2.3974151476 1.9333436963

H 4.0257812882 3.9777626021 -1.9475879205

H 1.3107023716 -0.6369910922 4.3580152755

H 0.2439688480 0.7124211434 4.4874984424

H -4.7129667494 4.2716161182 2.8569032326

H -6.4146086836 0.7362814317 1.1752670020

H 6.7812429031 1.2063061801 1.0436629239

H 5.9726135961 2.7817798021 -2.8356610663

H -6.5012109436 3.1661628711 1.5557428143

H 7.7197375536 1.3582829712 -2.3360453347

*Conformer mic-I*

O 0.5533649492 -3.9310217027 -1.5266966778

O -0.6911567737 -1.1086094226 -0.0608546676

O -1.4577469816 -3.1200736943 1.9909662082

O 2.3955091816 -2.5063732825 2.2633594427

O -3.8082568565 1.7846945701 2.5962658889

O 6.7650871684 1.9332859452 -0.5089161627

N -1.9206477129 -3.5850125122 -0.1707600460

N 2.0750749021 -1.6307258318 0.2052299914

N 1.0245653937 -4.3351068804 0.6433855522

N -2.6231045511 -0.1861312723 0.6570980582

N -3.9051113646 1.2105694770 -2.9032956444

N 4.7502481246 0.7013177737 -2.4352338079

N 0.3112462270 -0.6187836467 3.0102918107

N -1.8380258716 1.1778454439 3.5194380378

C -2.7182193651 -2.3841817592 -0.3480539686

C 2.4802866526 -2.8151443075 -0.5354316083

C -3.1126722440 -2.2278252160 -1.8250863805

C 2.9039197483 -2.4644193835 -1.9711360238

C -3.6796376426 -0.8742103603 -2.1067302089

C 3.4045082825 -1.0685040881 -2.1422136802

C -0.2612547647 -4.9541079488 0.9431006220

C 1.2613826505 -3.7563870520 -0.5411251610

C -4.8702415208 -0.3056472410 -1.5552599169

C 2.5927231612 0.0925438660 -2.3333919730

C -1.9051714537 -1.1642679414 0.0997091644

C -1.2782512077 -3.8014932723 0.9861629087

C -4.9698032417 1.0087086889 -2.0766672033

C -2.0508197357 1.0637914192 1.1135314309

C 3.4794514922 1.1843655371 -2.5052960606

C -3.1325972273 0.0843832151 -2.9111215699

C 4.7004813708 -0.6428551243 -2.2002592560

C 1.4341734657 -0.3026022208 2.1291267819

C 2.0198694464 -1.5805446262 1.5364310782

C -0.1883523364 -5.6933891795 2.2668820220

C -2.3451185736 2.2042280936 0.1261726521

C -5.8530092197 -0.7659591308 -0.6760557574

C 1.2141996895 0.3118479231 -2.3889435749

C 2.5179950343 0.4482420480 2.9157690592

C -6.0114825432 1.8586315499 -1.7244971333

C 3.0121440704 2.4755857562 -2.7216092299

C -1.5941116464 3.4405116335 0.5307071725

C -2.6549811303 1.3795141874 2.4904890922

C -6.8816420291 0.0815343941 -0.3325198151

C 0.7623055390 1.5941526876 -2.6035597498

C -6.9584752626 1.3796802816 -0.8462586180

C 1.6485711067 2.6645578229 -2.7646344233

C 3.6560842785 0.8360035294 2.0165232506

C -2.2363217911 4.4789522136 1.1910506672

C -0.2311722466 3.5436321634 0.2768789159

C 3.5623766010 1.9576820888 1.2010925260

C 4.8128592754 0.0719953497 1.9658032458

C -1.5317403523 5.6045438373 1.5827407282

C 0.4725194517 4.6700691289 0.6632134019

C 4.6032923495 2.3191423591 0.3712325171

C 5.8621317214 0.4221580406 1.1361375531

C -0.1765593824 5.7039994957 1.3173073872

C 5.7638680579 1.5526239091 0.3366199296

H -3.6035039702 -2.4630557158 0.2909351097

H 3.2943257484 -3.2816084054 0.0288241228

H -3.8350303224 -3.0067945232 -2.0770801256

H -2.2168491766 -2.3686540579 -2.4344825143

H 3.6668782811 -3.1753846501 -2.2880056073

H 2.0263133503 -2.6049717696 -2.6089339087

H -0.5092662695 -5.6296600315 0.1187194865

H -1.5229108233 -3.9979565698 -1.0076370249

H 1.5981238026 -0.9189466815 -0.3314099173

H 1.5776505922 -4.0272899834 1.4416271089

H -0.9734848025 0.9151015687 1.1980122598

H -2.2364158122 0.0330037112 -3.4995565482

H 5.5995268665 -1.2206009891 -2.1114982658

H -3.6297776627 -0.2593160548 0.7084610957

H 1.0668597392 0.3258920614 1.3122794511

H -3.7178481157 2.0529307067 -3.4199893092

H 5.5864832634 1.2625987739 -2.4513018740

H 0.6207459242 -6.4166173011 2.2467326874

H -0.0185425429 -4.9809687764 3.0711137854

H -1.1265439840 -6.2073277594 2.4527928500

H -3.4210071066 2.3893652793 0.1252072526

H -2.0338055763 1.8770835977 -0.8672076039

H -5.8135632165 -1.7697532574 -0.2786690988

H 0.5162870854 -0.5051042666 -2.2632403109

H 2.0620644352 1.3319087193 3.3626677281

H 2.8777319794 -0.2074879662 3.7112850930

H -6.0729900290 2.8597111464 -2.1225777697

H 3.6957804094 3.2999777663 -2.8545358087

H -7.6479848340 -0.2566896282 0.3490960608

H -0.3001701252 1.7855439197 -2.6501446020

H -7.7790958238 2.0137205849 -0.5466678350

H 1.2487840164 3.6548507118 -2.9238514502

H -0.3332678514 -1.2500743861 2.5358125164

H 0.6629121151 -1.1290180006 3.8166937608

H -3.2919678068 4.3965810094 1.4059178069

H 0.2779892522 2.7410444467 -0.2379887518

H 2.6664469560 2.5593563401 1.2262967061

H 4.8958698092 -0.8104167123 2.5838903492

H -2.2039503144 1.3169927030 4.4491134437

H -0.9546167637 0.6678432690 3.4053006805

H -2.0431665039 6.4058235999 2.0948354102

H 1.5287610070 4.7436113492 0.4498631153

H 4.5377117178 3.1949305407 -0.2541324738

H 6.7563880791 -0.1836643185 1.1140040371

H 0.3713835369 6.5837238596 1.6190438710

H 7.5680950575 1.4212608107 -0.3314195551

*Conformer mic-II*

O 1.2449442048 0.2272157145 -1.5107569836

O -3.7128779976 -1.9640896920 2.6834082688

O -2.0418943543 -3.2469426485 -1.1400260699

O 0.0955585121 0.6677408753 1.2306364962

O -0.9548341607 1.3459878404 4.7525607338

O -0.3340119881 5.5278874989 -3.8517489518

N -1.5198490927 -1.3730838118 0.0097152557

N 2.2505785922 1.2289197224 0.9543184623

N 1.1430216190 -1.7669864510 -0.4549837541

N -1.7754997343 -0.8095438509 2.7277476797

N -3.4176718232 0.5139029810 -3.3896023394

N 5.3857279735 -3.4539733675 -0.4234570735

N 1.9268680078 3.7387768675 1.6350316398

N -3.0492741059 0.6556390942 5.2093270219

C -2.8272914887 -1.1497469723 0.5838770756

C 2.6256479907 -0.0753395140 0.4460104765

C -3.3108178500 0.2787419790 0.3041327708

C 4.0207488148 -0.0328333939 -0.1935429328

C -3.3772313366 0.6190436316 -1.1504504848

C 4.4732565612 -1.4091426869 -0.5574631272

C 0.2018858088 -2.3765808004 -1.3666642999

C 1.5943728996 -0.5127312587 -0.6004983206

C -3.4867436650 1.9482367722 -1.6654868032

C 4.2110631731 -2.1013546359 -1.7791255845

C -2.8151440276 -1.3612283127 2.1013589844

C -1.2329850301 -2.3864979944 -0.8097826004

C -3.5040259694 1.8377086689 -3.0774232300

C -1.5459607164 -0.9236895251 4.1542427356

C 4.8052062918 -3.3825083508 -1.6546836872

C -3.3421114131 -0.2123727415 -2.2318044748

C 5.1851959453 -2.2711154072 0.2287391254

C 0.7733245662 2.8920028839 1.8914849662

C 1.0029436775 1.5055590006 1.3065705207

C 0.6449641678 -3.7859261953 -1.7374541610

C -0.1024735407 -1.3540920544 4.4335138330

C -3.5803847767 3.2090625185 -1.0767637762

C 3.5538049194 -1.7637626089 -2.9629558909

C -0.5807042699 3.4712825561 1.4157262302

C -3.5975188096 2.9578886687 -3.8947941060

C 4.7445953910 -4.3191168470 -2.6800326182

C 0.4126525823 -2.4320468978 3.5193014043

C -1.8111111405 0.4716650844 4.7493875798

C -3.6764131029 4.3130854408 -1.8923295058

C 3.4985412087 -2.6966372026 -3.9739604919

C -3.6801996609 4.1906156730 -3.2845683477

C 4.0857591405 -3.9589359459 -3.8352677270

C -0.5214462871 3.9968350674 0.0079626675

C -0.3994787962 -3.4554475139 3.0440936226

C 1.7521053217 -2.4192443842 3.1465065453

C -0.6317362667 5.3601309940 -0.2406550614

C -0.3440862276 3.1430877411 -1.0740764056

C 0.1128068613 -4.4317455630 2.2063176822

C 2.2691993918 -3.3972433080 2.3134414248

C -0.5730079225 5.8601429073 -1.5262086866

C -0.2740532353 3.6322121877 -2.3633815111

C 1.4478183354 -4.4079655958 1.8387490770

C -0.3922344685 4.9958367125 -2.6006691184

H -3.5115099695 -1.8856034258 0.1493012420

H 2.6175260676 -0.7952342665 1.2730774880

H -2.6241046219 0.9771679145 0.7912562917

H -4.2962749201 0.4048361100 0.7628519172

H 3.9765792219 0.5960162251 -1.0844148774

H 4.7168077129 0.4070266839 0.5216397085

H 0.1774077318 -1.7392211041 -2.2611376948

H -0.8184347406 -0.6646849143 0.1929080176

H 2.9303766212 1.9763702172 1.0295132525

H 1.4434426178 -2.3303624616 0.3295749029

H -2.2727115767 -1.6434467884 4.5411415938

H -3.2668350904 -1.2821893111 -2.2679971511

H 5.5805362466 -2.1129179343 1.2134623432

H -1.0842292283 -0.2736850630 2.2104750654

H 0.6956098358 2.7250686356 2.9754335493

H -3.4075919866 0.1258593326 -4.3169786881

H 5.9009857569 -4.2396296034 -0.0643760161

H 1.6130430824 -3.7595075003 -2.2290315996

H 0.7058429409 -4.3981704998 -0.8421845808

H -0.0906968428 -4.2271915117 -2.4029157436

H -0.0401669674 -1.6926813674 5.4708436508

H 0.5295210205 -0.4671721204 4.3373190677

H -3.5794338411 3.3153964748 -0.0023900030

H 3.0889793807 -0.7955904013 -3.0790838919

H -1.3218700968 2.6726685936 1.4895198805

H -0.8773068554 4.2727499152 2.0941280828

H -3.6092039117 2.8635377834 -4.9701687499

H 5.2000296077 -5.2920280418 -2.5773013955

H -3.7451911571 5.2978539020 -1.4546226789

H 2.9913876830 -2.4554890268 -4.8968874959

H -3.7481767351 5.0831516418 -3.8886384268

H 4.0187832202 -4.6603702177 -4.6534185099

H 1.8810134744 4.5639178008 2.2212871560

H 1.8939424874 4.0572796954 0.6695558073

H -1.4415825830 -3.5014658086 3.3217773463

H 2.3950667930 -1.6387662137 3.5271724487

H -0.7742778176 6.0445873639 0.5831304532

H -0.2528915543 2.0785269010 -0.9222786483

H -3.3195382018 1.5671802167 5.5465672248

H -3.7490638994 -0.0624048197 5.1231168548

H -0.5379225674 -5.2124318682 1.8407525195

H 3.3117287529 -3.3728583633 2.0338448255

H -0.6665860202 6.9170622998 -1.7198364541

H -0.1350782510 2.9435509637 -3.1838448006

H 1.8486125237 -5.1765404205 1.1953714194

H -0.1532513138 4.8343532768 -4.5028720071

*Conformer mic-III*

O 0.3924284452 -4.0102947968 -1.5849828195

O -0.7836244213 -1.0915998407 -0.0818876192

O -1.3818530223 -3.2022471353 2.0597224149

O 2.4386016566 -2.6092938516 2.1213397356

O -4.3723223380 1.0384761477 2.3327368324

O 6.6661855965 2.2048614700 -0.6685036048

N -1.9558649333 -3.6623054664 -0.0761225256

N 1.9729242230 -1.6869006963 0.1109431270

N 1.0119627714 -4.4099536527 0.5504116889

N -2.6721784499 -0.3319151561 0.8946427426

N -3.8692002981 1.1532126848 -2.7916262579

N 5.0030087589 0.3756269976 -2.5317428297

N 0.4351435826 -0.6797564586 3.0438709864

N -2.8170945642 2.4568972684 3.1372393422

C -2.7673673594 -2.4665481100 -0.2194476719

C 2.3337635638 -2.8341290944 -0.7068218528

C -3.1921216397 -2.3007872876 -1.6872410928

C 2.5904947284 -2.3934653005 -2.1531993094

C -3.7099426997 -0.9268565029 -1.9663661378

C 3.3456052245 -1.1103123423 -2.2602892587

C -0.2451841656 -5.0366247669 0.9425315396

C 1.1533208319 -3.8168729426 -0.6429683487

C -4.8622046171 -0.3020338771 -1.3953293977

C 2.7825047605 0.2035019995 -2.2445334693

C -1.9671500383 -1.2315760723 0.2146039434

C -1.2581612023 -3.8834398182 1.0463925544

C -4.9215712830 1.0068496020 -1.9362021650

C -2.1862337449 0.9527068266 1.3446028483

C 3.8611935591 1.1074449993 -2.4100448303

C -3.1439513670 -0.0040400988 -2.7988033385

C 4.6920029409 -0.9490031087 -2.4217200607

C 1.5078237906 -0.3864136287 2.0988787640

C 2.0211536958 -1.6630914775 1.4423430898

C -0.0757699180 -5.7649840212 2.2630741478

C -2.0521064695 1.9117218544 0.1392968723

C -5.8388047330 -0.7067897338 -0.4837287380

C 1.4841312019 0.7047715458 -2.1219732606

C 2.6579314628 0.3114615692 2.8428727365

C -5.9168449932 1.9051847042 -1.5698573608

C 3.6616329485 2.4822317145 -2.4460963750

C -1.4557622980 3.2400631000 0.5001312665

C -3.2310184833 1.4857320971 2.3271739456

C -6.8212892797 0.1877216095 -0.1263608914

C 1.2980853457 2.0684935348 -2.1500694484

C -6.8580864554 1.4797034984 -0.6583979298

C 2.3735684012 2.9478372859 -2.3055446268

C 3.7078675754 0.8247997578 1.9009302054

C -2.2560984881 4.3729948534 0.5846786321

C -0.0912557663 3.3605049103 0.7412980874

C 3.7216147821 2.1546363933 1.5060583428

C 4.6934819003 -0.0223523720 1.4082469925

C -1.7055315786 5.6034701511 0.8997141308

C 0.4599091438 4.5910516530 1.0514987994

C 4.6989602145 2.6375034061 0.6561984731

C 5.6722958404 0.4450866885 0.5559516431

C -0.3450851803 5.7153710761 1.1320639211

C 5.6835930923 1.7837898902 0.1778459245

H -3.6382766493 -2.5609179476 0.4369056593

H 3.2192168125 -3.2944075554 -0.2554652050

H -3.9491420062 -3.0523443909 -1.9200608258

H -2.3157138798 -2.4772510626 -2.3157080239

H 3.1294645196 -3.1940908194 -2.6608802538

H 1.6168307873 -2.2852534463 -2.6380268509

H -0.5473109860 -5.7189878110 0.1422431686

H -1.6083150696 -4.0836469387 -0.9312698261

H 1.4632056311 -0.9559201893 -0.3655032046

H 1.6101141849 -4.0867285908 1.3107907488

H -1.2193297067 0.8270886095 1.8416001258

H -2.2634721895 -0.0991363985 -3.4048135818

H 5.4548059857 -1.7004302165 -2.4868650694

H -3.6596708711 -0.4712111766 1.0896970208

H 1.1042961086 0.2717561961 1.3228527293

H -3.6607006455 1.9758562855 -3.3312384812

H 5.9348837385 0.7545921130 -2.5740393360

H 0.1491146210 -5.0449023679 3.0469915905

H -0.9969470951 -6.2790965034 2.5199531183

H 0.7324952667 -6.4858908002 2.1913744534

H -3.0460869964 2.0451356044 -0.2918653113

H -1.4192873273 1.4041435828 -0.5922562516

H -5.8281858637 -1.7042004837 -0.0689993527

H 0.6377832211 0.0379416568 -2.0137185713

H 2.2297349696 1.1276834949 3.4241718204

H 3.0965824514 -0.4161070410 3.5303525064

H -5.9478942656 2.9017800285 -1.9834990198

H 4.4894285761 3.1586244627 -2.5890758529

H -7.5788214172 -0.1068197557 0.5844156650

H 0.3033816757 2.4783200375 -2.0547367572

H -7.6424139420 2.1525072294 -0.3460936807

H 2.1800139526 4.0097468601 -2.3206511103

H -0.2833583523 -1.2430236622 2.5930459195

H 0.8105094839 -1.2536435544 3.7944198124

H -3.3166066274 4.2869044787 0.3977321894

H 0.5432647834 2.4891632294 0.6670081210

H 2.9635051374 2.8250135001 1.8808646000

H 4.7004174940 -1.0607232114 1.7069750667

H -3.4698020825 2.8643172335 3.7887595340

H -1.8719516540 2.7980743404 3.1037296405

H -2.3380678662 6.4760714530 0.9579586729

H 1.5226441337 4.6757801467 1.2207065187

H 4.6960681778 3.6778252330 0.3657597440

H 6.4428751162 -0.2113185200 0.1838742893

H 0.0868078797 6.6752738711 1.3702880153

H 6.6518456800 3.1705525737 -0.7433782068
